# Polyhexanide-Releasing Membranes for Antimicrobial Wound Dressings: A Critical Review

**DOI:** 10.3390/membranes12121281

**Published:** 2022-12-18

**Authors:** António Jorge Guiomar, Ana M. Urbano

**Affiliations:** 1 Chemical Process Engineering and Forest Products Research Centre, Department of Life Sciences, University of Coimbra, 3000-456 Coimbra, Portugal; 2 Molecular Physical-Chemistry R&D Unit, Center of Investigation in Environment, Genetics and Oncobiology-CIMAGO, Department of Life Sciences, University of Coimbra, 3000-456 Coimbra, Portugal

**Keywords:** poly(hexamethylene biguanide), polyhexamethylene biguanide, polyhexanide, PHMB, membrane, controlled drug release, wound dressing, antimicrobial, cytotoxicity, wound healing

## Abstract

The prevalence of chronic, non-healing skin wounds in the general population, most notably diabetic foot ulcers, venous leg ulcers and pressure ulcers, is approximately 2% and is expected to increase, driven mostly by the aging population and the steady rise in obesity and diabetes. Non-healing wounds often become infected, increasing the risk of life-threatening complications, which poses a significant socioeconomic burden. Aiming at the improved management of infected wounds, a variety of wound dressings that incorporate antimicrobials (AMDs), namely polyhexanide (poly(hexamethylene biguanide); PHMB), have been introduced in the wound-care market. However, many wound-care professionals agree that none of these wound dressings show comprehensive or optimal antimicrobial activity. This manuscript summarizes and discusses studies on PHMB-releasing membranes (PRMs) for wound dressings, detailing their preparation, physical properties that are relevant to the context of AMDs, drug loading and release, antibacterial activity, biocompatibility, wound-healing capacity, and clinical trials conducted. Some of these PRMs were able to improve wound healing in in vivo models, with no associated cytotoxicity, but significant differences in study design make it difficult to compare overall efficacies. It is hoped that this review, which includes, whenever available, international standards for testing AMDs, will provide a framework for future studies.

## 1. General Introduction and Aims

Wounds can be viewed as a disruption of the normal anatomical structure and function of any bodily tissue or organ. They can occur following injury by physical, chemical or thermal means, or as the result of underlying pathological conditions. In open wounds, the protective body surface (skin or mucous membranes) is broken, allowing entry of foreign material into the tissues, including microorganisms. Wound healing, i.e., the process by which the normal structure and function of destroyed or damaged tissues is restored, is an extremely complex and dynamic biological process that can be divided into the following four sequential, yet overlapping, processes: (i) hemostasis, i.e., stoppage of bleeding; (ii) inflammation, a primary defense mechanism against the invasion of microorganisms; (iii) proliferation of keratinocytes, fibroblasts, macrophages and endothelial cells and migration from the wound edges into the gap in the dermal layers created by the wound, to restore skin continuity and function through the formation of new tissues and blood vessels; (iv) remodeling of these new tissues, converting the initial fibrin-rich blood clot into a collagen-rich scar [1,2]. As a result of the inflammatory process, capillaries become more permeable, allowing entry into the wound bed of blood cells and fluid (the so-called exudate).

Attenuation or disruption of any of the cellular or molecular mechanisms underlying the above-mentioned sequence of processes can compromise wound closure, with wound healing usually stalling in the inflammatory phase. Advanced age and diabetes are primary risk factors for developing chronic, non-healing wounds, but failure to achieve wound healing in a timely and orderly manner may also be due to several other factors, such as other pathological conditions (e.g., malignancies), repeated insults, poor primary treatment, necrosis and excessive levels of inflammatory cytokines and exudate. Chronic wounds caused by pathological conditions, also known as ulcers, have a high prevalence worldwide, most notably venous leg ulcers and diabetic foot ulcers, for which the worldwide prevalence is 3% and 6.3% of the adult population, respectively [3].

Chronic wounds are often severely colonized by bacteria and fungi and may become infected. The formation of biofilms in polymicrobial infections shields bacteria from antimicrobial agents and from the immune response and is one of the causes of wound-healing failure, which can result in the widening of wounds, in the need for surgical intervention and in life-threatening events [4]. In the fight against wound infection, the most common approaches involve the removal of dead, damaged and/or infected tissue from the wound (wound debridement) and their cleansing with topical antiseptics, such as the oxidizing agents hypochlorous acid and hydrogen peroxide, and the polymer-based povidone-iodine (PVP-I) and poly(hexamethylene biguanide), also known as polyhexanide and abbreviated as PHMB [5].

The simplest approach to replace the barrier function of intact skin is the application of a dressing, such as cotton gauze. Ideally, traditional wound dressings (WDs) should possess the following characteristics [6,7]: (i) stand as a temporary barrier against microorganisms; (ii) protect from trauma; (iii) possess mechanical stability; (iv) absorb excess exudate or provide hydration, depending on the wound characteristics; (v) allow for gas transmission; (vi) relieve pain; (vii) have low adherence to skin, causing minimal pain during application and removal; (viii) be non-toxic and non-irritant; and (ix) be sterile. Aiming at a more active role in wound healing, some WDs, known as medicated (or bioactive) wound dressings, have a bioactive agent incorporated into their matrices, most commonly antimicrobials, but also analgesics, anesthetics, anti-inflammatory drugs and growth factors. WDs and medicated WDs have been in use since antiquity [8,9], with some of our early ancestors covering wounds with dressings made from locally available herbs and natural fibers, favoring those that accelerated healing. For instance, the Egyptians employed antimicrobial dressings (AMDs) in the form of plasters (adhesive bandages) made of honey, grease and lint and applied a malachite-containing “green paint” to wounds. It is now known that honey, which is still employed in commercial AMDs [10], has antimicrobial properties, due to the presence of hydrogen peroxide, polyphenols, phenolic acids, and flavonoids. Grease has barrier properties, lint may have helped in exudate drainage and malachite is a mineral rich in copper, a metal known for its antimicrobial properties. For the Greeks, the importance of cleanliness was introduced and washing of wounds with boiled water, vinegar and wine was common practice, due to the antiseptic action of vinegar and wine conferred by acetic acid and ethanol, respectively. In the 20th century, the introduction of antibiotics significantly improved the control of wound infection, but its widespread use led to the appearance of multidrug-resistant bacterial strains. The use of antiseptics is now favored, particularly those with an unspecific mode of action, i.e., whose antimicrobial activity is due to the irreversible destruction of the cell membrane or of the bacterial cell itself, or to the blockage of negative surface charges, as no resistance associated with their normal use has been reported [5,11,12]. In addition, the risks for contact sensitization and systemic effects are lower. Nonetheless, it must be stressed that no wound dressing that contains antiseptics should be used routinely, but only when signs of wound infection exist, with the exception of those patients for whom prophylactic use is advised, namely immune-compromised patients [13].

The WD market is expected to represent USD 11.2 billion by 2025 [14], driven mostly by the aging population, the steady rise in diabetes and obesity, but also the increasing numbers of road accidents and surgeries, including caesarian sections in older women, among other factors. However, to this day, and in spite of intensive research efforts, the number of AMDs that have reached the market has been limited, due to a combination of high production costs, poor drug stability, demanding storage conditions [15] and difficulties in achieving a drug delivery system in which the full therapeutic effect of the loaded bioactive agents is safeguarded [16]. Moreover, many wound-care professionals agree that none of the commercially available AMDs show optimal or comprehensive antimicrobial power [17].

Of the commercially available AMDs, more than a dozen contain the antiseptic PHMB. Among the advantages of using PHMB in AMDs are its high stability [18,19] and broad antibacterial spectrum. Among the antiseptics evaluated in a recent consensus report on wound antisepsis, PHMB was considered as the first choice for burns and for critically colonized and infected chronic wounds and one of two first choices for the treatment of contaminated acute and chronic wounds [5]. Here, research on PHMB-releasing membranes (PRMs) designed and tested for application as AMDs will be reviewed for the first time. Besides describing their preparation and characterization, this manuscript also presents international standards that might be used as guidance in the analysis of their performance, as well as in the design and testing of novel studies and/or PRMs. For contextualization, the main discussion is preceded by a brief overview of the methods employed in the preparation of polymer membranes for WDs, the main characteristics of PHMB and the commercially available PHMB-releasing wound dressings (PRWDs). Antimicrobial membranes and WDs in which PHMB is covalently attached to the matrix and, as a consequence, is not released (e.g., [20]), are beyond the scope of this review.

## 2. Preparation of Selective Polymer Membranes for Wound Dressings

**Figure 1:**
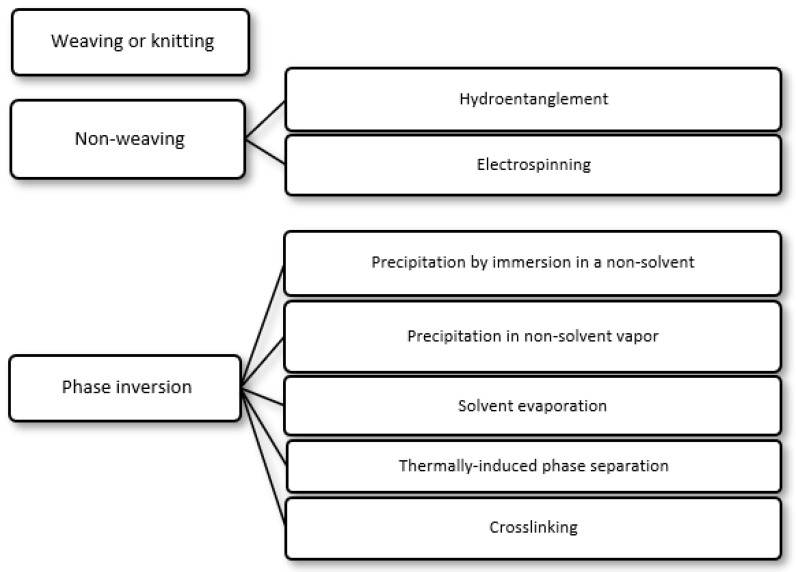
Methods commonly employed in the preparation of membranes for wound dressings.

The matrices of commercially available WDs of known composition are all made of one or more natural polymers, modified natural polymers or synthetic polymers. These polymeric matrices, which commonly have the form of a sheet or pad, act as selective membranes, controlling mass transport of a particular species across them. In particular, they prevent the transport of microorganisms and other foreign agents present in the outside environment to the wound, while allowing transport of water vapor, oxygen and carbon dioxide to or from the outside environment. Membranes for WDs are most commonly produced by the following methods (Figure 1): (i) weaving or knitting, (ii) non-weaving and (iii) phase inversion. In weaving, a fabric-like structure is achieved by interlacing two distinct sets of fiber yarns at right angles, with the aid of a weaving machine; in knitting, the yarn interlacing pattern is more elaborate, achieved by yarn interlocking [21]. In non-weaving, fabrics are made directly from the polymer or from its fibers, without the need for yarn. The methods most commonly employed in the production of non-woven fabrics for WDs are hydroentanglement (also known as spunlacing) and electrospinning. In hydroentanglement, fibers are entangled as a result of the action of a curtain of fine, high-pressure water jets that penetrate fiber webs and hit a conveyor belt below the fiber webs, bouncing back through the fiber webs [22]). In electrospinning, a polymer solution or melt is forced through a narrow needle in a high-voltage electrical field created between the needle and a collector surface. This solution or melt is, thus, converted into a charged polymer jet that is accelerated towards the collector by the electrical field. Solvent evaporation occurs and a non-woven web composed of fibers with diameters in the nanometer range is deposited on the collector’s surface [23]. In addition, a natural non-woven membrane of cellulose is produced by bacteria such as *Acetobacter xylinum* (*A. xylinum*) and designated bacterial cellulose [24]. Currently, non-wovens are favored in relation to wovens, since they are faster and more economical to produce [21,25]. In phase-inversion methods, a polymer solution, composed of a solvent (continuous phase) and a polymer (non-continuous phase), is converted into a polymer membrane, commonly after casting on a surface [26,27]. In the resulting membrane, the component phases are inverted with respect to the polymer solution, with the polymer becoming the continuous phase, and the solvent in the membrane’s pores becoming the non-continuous phase. Phase inversion can be accomplished by several methods, including the following (Figure 1):

(i) Precipitation by immersion in a non-solvent, in which precipitation occurs due to the exchange of the solvent in the polymer solution by a non-solvent miscible with the solvent present in a coagulation bath;
(ii) Precipitation in non-solvent vapor, in which phase inversion occurs inside a closed chamber in which the non-solvent is in the vapor phase and causes precipitation of the polymer in the solution;
(iii) Solvent evaporation (also known as solution casting), in which the solvent of a polymer solution cast on a substrate is allowed to evaporate or is removed by heating the polymer solution in an oven;
(iv) Thermally induced phase separation, in which a solvent that dissolves the polymer at a given temperature loses this ability when the temperature is decreased, with the solvent being subsequently removed by extraction, evaporation or lyophilization (freeze-drying);
(v) Crosslinking, in which a polymer in solution is insolubilized by the formation of chemical bonds or by physical interactions between its molecular chains.

## 3. The Antiseptic Polyhexanide (PHMB)

PHMB is a synthetic polydispersed mixture of polymers that has been widely employed as an antiseptic since the 1950s. In addition to its use in wound antisepsis [5], PHMB has found a wide range of other applications, including the following: (i) disinfection of medical utensils and trays, food and non-food contact surfaces, animal drinking water, recreational water, filters and toilets, (ii) preservative in contact lens solutions, cosmetics and personal care products, wet wipes, fabric softeners, and hand and mouth washes, (iii) preservation of hides and skins, (iv) anti-odor finishes in textiles and (v) antimicrobial high-pressure paper–phenol-formaldehyde resin laminates [28,29,30]. It has also found other types of applications, such as in gene delivery [31], DNA capture for biological threat surveillance [32], antibiofouling filtration membranes [33], dental-plaque control [34], cotton fabric dyeing [35], fuel cells [36], optoeletronics [37], CO_2_ capture and sensing [38], uranium extraction from seawater [39] and sewage dewatering [40]. As a preservative in cosmetic products, it has been considered by the European Commission’s Scientific Committee on Consumer Safety (SCCS) as safe for consumers up to a concentration of 0.1% [28].

PHMB is effective against a wide spectrum of both Gram-positive and Gram-negative bacteria, including difficult to control bacterial strains, such as methicillin-resistant *Staphylococcus aureus* (MRSA) and vancomycin-resistant *Enterococci* (VRE) [41], although a single polyhexanide decolonization course may not be sufficient to eradicate MRSA in clinical cases [42]. Of note, no PHMB-resistant MRSA could be isolated in clinical cases in which decolonization by PHMB was attempted [42]. On the contrary, decreased susceptibility to PHMB could be artificially induced in vitro by repeated exposure to very low PHMB concentrations (0.1–1 µg/mL or 0.00001–0.0001%). This reduced susceptibility was associated with specific genomic alterations [43]. PHMB is also active against yeasts and other fungi [44], amoeboids such as *Acanthamoeba* spp. [45], enveloped viruses, such as *Herpes simplex* [46] and human immunodeficiency virus type I (HIV-I) [47], and non-enveloped viruses [48].

PHMB’s minimum inhibitory concentration (MIC) and minimum bactericidal concentration (MBC) values against bacteria commonly found in wounds are presented in Table 1. As can be observed, all the values are in the low µg/mL range, but there are some significant discrepancies between the values reported by different laboratories, which might be due, at least partially, to the use of different strains. Within the same study, the lowest values consistently corresponded to the Gram-negative bacteria *Staphylococcus aureus* (*S. aureus*) and *Escherichia coli* (*E. coli*), and the highest to the Gram-positive bacteria *Pseudomonas aeruginosa* (*P. aeruginosa*). MIC and MBC values may be higher when bacteria form biofilms, as suggested by the results of a study that show that the antimicrobial activity of a commercial PRWD was higher in a planktonic cell model than in an immobilized cell model used to mimic biofilms [49]. PHMB is well tolerated when administered topically to wounds, showing low cytotoxicity and poor skin penetration [28,44,50], and also promotes wound healing, by favoring tissue granulation on the wound surface [5]. In humans, repeated prolonged exposure to PHMB at concentrations of 2% may cause sensitization [51], most notably in patients with suspected allergic contact dermatitis [52]. However, this PHMB concentration is 4-times higher than the highest concentration employed for antisepsis. For instance, the wound irrigation/cleaning solution Serasept^®^ (Serag-Wiessner GmbH & Co. KG, Naila, Germany) contains PHMB at a concentration of 0.03% or 0.04%, and Prontosan^®^, in all its three formats (solution, gel or spray; B. Braun Melsungen AG, Germany), contains 0.1% PHMB (in combination with a betaine antiseptic). In rats, acute skin toxicity was only observed for a concentration of 5% [44].

**Figure 2:**
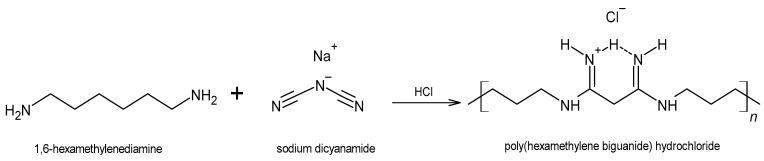
Synthesis of poly(hexamethylene biguanide) hydrochloride (PHMB.HCl) via polycondensation of 1,6-hexamethylenediamine and sodium dicyanamide. *n* = 2–42.

**Figure 3:**
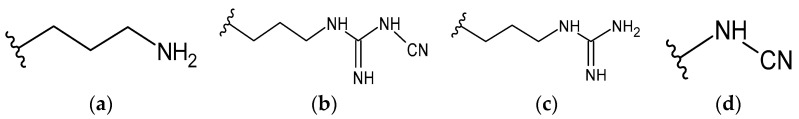
Terminal groups that may occur in poly(hexamethylene biguanide) (PHMB) chains. (**a**) Amino; (**b**) cyanoguanidino; (**c**) guanidino; and (**d**) cyanoamino.

PHMB is commonly synthesized as a hydrochloride salt by polycondensation of 1,6-hexanemethylenediamine and sodium dicyanamide, but can also be synthesized through other routes (Figure 2). The polycondensation process generates a mixture of oligomers, composed of a minimum of 2 and a maximum of 40 to 42 repeating units, with an average degree of polymerization of 12. The oligomer chains can have different terminal groups, including guanidino, cyanoguanidino, amino and cyanoamino groups (Figure 3) [56,57,58].

Due to the occurrence of polymerization, depolymerization and repolymerization reactions during its synthesis, a polydisperse polymer is obtained. Reported molecular weight (MW) values range from below 500 g/mol up to ca. 4200 g/mol [28], and MW theoretical estimates point to ca. 1400 g/mol for its number-average MW and ca. 2600 g/mol for its weight-average MW [58]. Of note, a positive correlation was observed between polymer chain length and antiseptic/antimicrobial activity [4]. PHMB is structurally related to chlorhexidine (CHX), another synthetic biguanide-based antiseptic that possesses two biguanide units linked by a hexamethylene chain, with 4-chlorophenyl as the terminal group.

PHMB is also structurally related to naturally occurring antimicrobial peptides (AMPs) [41], which similarly exhibit a broad spectrum of activity against bacteria, viruses and fungi. These peptides are part of the innate immune response, being produced by cells within wounds, such as keratinocytes and neutrophils [56]. Chemically, AMPs and PHMB can both be described as polycations, the former containing positively charged amino acids among a larger proportion of hydrophobic amino acids, the latter containing repeating biguanide units of high basicity (calculated p*K*_a_ values above 13 [59]), attached to flexible hydrophobic hexamethylene chains (Figure 2). The mechanisms of action of PHMB and AMPs are also related, both relying upon their binding to the negatively charged bacterial cell membranes and walls, ultimately inducing cell lysis through the disruption of membrane integrity. In Gram-positive bacteria, this negative electrical charge is due to the presence of anionic phospholipids, teichoic acids and polysaccharides, whereas it is due to lipopolysaccharides in Gram-negative bacteria. In PHMB, the positively charged biguanide units are able to displace Ca^2+^ ions that stabilize the lipid bilayer and its hydrophobic hexamethylene portion is able to sink into the cell membrane, resulting in increased membrane permeability and, ultimately, membrane disruption [11,56,60,61]. Complexation with negatively charged phospholipids also impairs the function of ion pumps, receptors and enzymes in the cell membrane [56]. In addition, PHMB is able to enter the bacterial cell and arrest cell division by binding and condensing bacterial chromosomes [12]. The specificity of PHMB to bacterial cells can be partially explained by the relatively neutral surface of eukaryotic cells, due to the presence of phosphatidylcholine-based phospholipids and other zwitterionic lipids, as well as cholesterol. PHMB is still able to enter the eukaryotic cell, but no damage to the cell membrane occurs. Additionally, it does not penetrate the nucleus, being trapped within endosomes, which are absent in bacteria [12].

In terms of physical and chemical properties, PHMB exhibits good thermal and hydrolytic stability [18,19]. It is highly soluble in water and methanol (ca. 40%), but only sparingly soluble in ethanol (ca. 0.5%) and in some other organic solvents, such as acetone, acetonitrile, dichloromethane and toluene [28]. In water, but not in ethanol [58], the alternating sequence of hydrophilic (biguanide) and hydrophobic (hexamethylene) segments may give rise to polymeric micelles of the core/shell type, in which the hydrophobic segments point towards the center of a sphere (core), while the hydrophilic groups point outwards (shell). However, at the concentrations normally employed for wound antisepsis and commercial PRWDs (0.01–0.05% [62]), micelle formation is not expected, as PHMB’s critical micellar concentration (CMC) is considerably higher (0.02–0.05 M in water [58], equivalent to ca. 50–130 mg/mL or 5–13%). On the other hand, in water and at concentrations below its CMC, PHMB may form aggregates larger than the above-mentioned micelles, with particle dimensions in the micrometer range [58]. When in aqueous solutions of monovalent counterions, molecular dynamics simulation studies suggested that PHMB can self-assemble in a compact, ordered hairpin-like structure [63] of the type that occurs in biological polyelectrolytes, such as RNA [64]. With increasing salt concentration, these hairpin-like structures can collapse further into three- or five-folded structures. Neither ions nor water molecules mediate this self-assembly, which, counterintuitively, is driven by like-charge pairing of free biguanidinium ions [65].

## 4. Commercially Available PHMB-Releasing Wound Dressings (PRWDs)

A first proposal for the medical use of a PHMB-releasing membrane can be found in a 1984 patent filed by Johnson & Johnson [66] for the invention of a non-woven, cellulose-based PHMB-containing fabric to be used in surgical procedures as a means to isolate the surgical area from the rest of the body and other sources of contamination. This surgical drape was prepared by spraying the non-woven fabric with PHMB dissolved in a solution of a fabric binder. Approximately two decades later, PHMB was successfully introduced into wound management in the form of AMD. This success was due, at least partially, to PHMB’s high stability and broad antimicrobial spectrum [18,19]. In commercial PHMB-containing WDs, PHMB is either in its free form, being released over time into the wound and periwound tissues, or chemically bound to the wound dressing matrix. As mentioned at the end of the General Introduction and Aims section, the latter type will not be discussed in the present review.

Table 2 summarizes some of the characteristics of commercially available PRWDs. As can be observed, their matrices were made from a wide variety of polymers, either of natural origin (cotton, viscose, rayon, bacterial cellulose and extracellular matrix biopolymers) or synthetic (polyesters and polyurethanes (PUR)). Whenever this information is disclosed, PHMB loading was always by impregnation, employing PHMB solutions of concentrations ranging from 0.1% to 0.5%, and antibacterial activity lasted for up to 3–7 days. The biological properties of some of the PRWDs were assessed in reported studies. A full discussion of all of these studies is out of the scope of the present review. Instead, a very brief overview of the results obtained for the most studied commercial PRWDs will be presented, in the order of their appearance in Table 2. Indirect reference to some of their properties will also be made in Section 5, when discussing studies of PRMs in which commercial PRWDs were also tested for comparison purposes. Starting with ActivHeal^®^ PHMB, its employment in 32 patients with hard-to-heal wounds (such as post-operative surgical wounds and leg, diabetic and pressure ulcers) reduced bacterial load and pain and resulted in the effective management of exudate levels [67]. CelluDress-PHMB, a more recent addition to the market, was associated with good outcomes in the treatment of non-healing venous leg ulcers [68]. In clinical cases, the Excilon^TM^ AMD drain sponge extended the number of days during which tracheostomy sites remained free of pathogens, compared to a non-AMD dressing, while normal flora remained unaffected [69]. Kendall™ AMD foam dressing resisted in vitro MRSA colonization within the WD more efficiently than an equivalent, non-impregnated WD [70]. In vivo, this PRWD performed well in a small-scale (25 subjects), retrospective uncontrolled environment study, with improvement in all wounds [71], and its employment in a multicenter, prospective, double-blind, pilot, randomized controlled clinical trial that involved 45 patients with chronic wounds resulted in higher reductions in bacterial burden, pain and wound size than the employment of a similar, non-AMD, PUR-based WD (Kendall™ foam dressing) [72]. Kerlix™ AMD inhibited the in vitro proliferation, both within and underneath the WD, of Gram-negative and Gram-positive bacteria [73], including antibiotic-resistant bacterial strains, such as VRE [74] and MRSA [73,75]. In humans with difficult-to-heal wounds and in a wound-healing porcine model (Section 5.2.5), it decreased the bacterial burden, compared to a similar WD without PHMB, while sparing the normal skin flora (only assessed in the porcine model) [76,77]. In the same porcine model, no adverse effects on the wound epithelization rate were observed [78]. However, in another study that also employed a porcine model, enhancement or lengthening of the wound inflammatory response occurred, resulting in bleeding when changing the WD [79]. Suprasorb^®^ X + PHMB was assessed in case studies of patients with traumatic and surgical wounds, pressure and leg ulcers, and burns, where it reduced infection, exudate levels and pain, while increasing granulation tissue and epithelialization and improved necrotic tissue debridement [80]. In a co-culture of *S. aureus* and keratinocytes, Suprasorb^®^ X + PHMB protected keratinocytes from damage by reducing the number of viable bacterial cells [81]. In a prospective, randomized, controlled single-center study designed to compare its clinical efficacy with that of a silver-sulfadiazine cream and dressing in 60 patients with partial-thickness burns (i.e., second-degree burns), no difference in healing times was found, but Suprasorb^®^ X + PHMB showed better and faster pain reduction and the need for fewer WD changes [82]. Additionally, in a prospective, randomized, controlled, multicenter trial with 25 patients per treatment group, it was found that Suprasorb^®^ X + PHMB and a silver-containing WD were both effective in reducing pain and bacterial burden in the treatment of critically colonized and locally infected wounds, but Suprasorb^®^ X + PHMB exhibited superior antimicrobial properties [83]. Finally, Telfa™ AMD was assessed in a prospective, randomized controlled study that comprised 32 patients treated for skin burns with skin grafts. In this study, patients treated with this AMD exhibited shorter re-epithelialization times and lower pain levels than the patients treated with Bactigras^®^, a commercial AMD with the antiseptic CHX [84].

## 5. PHMB-Releasing Membranes (PRMs) for Antimicrobial Dressings (AMDs)

The first publications that reported the preparation and assessment of PRMs for AMDs date back to the early 2010s. Since then, a total of 28 PRMs have been described in the literature (Table 3). In the following sections, the composition and methods employed in their preparation will be detailed, followed by a discussion of their physical and biological properties. Table 4 summarizes the characterization studies carried out with each PRM.

### 5.1. Composition and Preparation Methods

A variety of polymers and polymer mixtures were employed in the preparation of the 28 PRMs. Some of these polymers, such as cotton, bacterial cellulose and polyurethanes (PURs), are also employed in the preparation of commercial PRWDs (Table 2), while others are unique to these PRMs, such as the natural-origin polymers chitosan, alginate, pectin, cellulose acetate (CA), wool, gelatin, silk sericin (SS), silk fibroin (SF) and fibrin, and the synthetic polymers poly(lactic acid) (PLA), poly(vinyl alcohol) (PVA), polyamide (PAm), polydimethylsiloxane (PDMS) and a poly(*N*-isopropylacrylamide)/*N*-tert-butylacrylamide/acryloyl-lysine (PNIPAAm/NtBAAm/A-Lys) copolymer. The latter case explores the thermally induced contraction of a PNIPAAm-based hydrogel to favor drug release at human body temperatures. This type of stimuli-reacting system is sometimes described as “smart”. Another interesting strategy involved the inclusion of a protease in a gelatin-based matrix to decrease the membrane’s crosslinking degree over time.

#### 5.1.1. Preparation of the Polymer Membrane

Some of the polymer membranes in PRMs were non-woven bacterial cellulose membranes produced by bacteria. The remaining membranes were prepared using a variety of methods (Table 3), mostly by phase inversion, non-weaving and weaving or knitting. Phase-inversion membranes were prepared by solvent evaporation, thermally induced phase separation by freeze-drying or by freeze-drying after freeze/thaw cycling, and by crosslinking. Non-woven membranes were prepared by electrospinning and, when the preparation method is mentioned, woven membranes were prepared by weaving. A different approach was the preparation of the polymer membrane from monomers, employing free-radical polymerization.

#### 5.1.2. PHMB Loading

**Figure 4:**
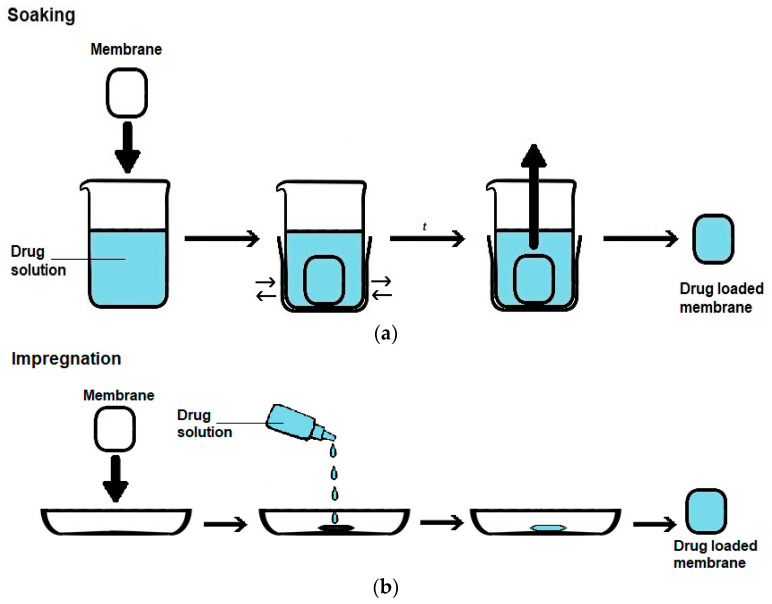

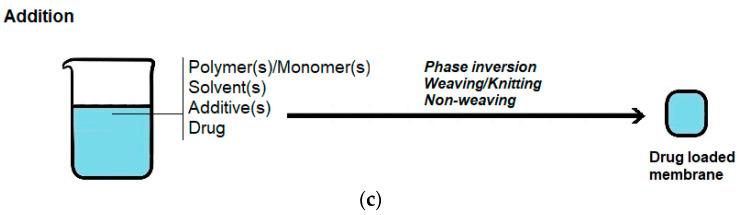
Schematic representation of the methods employed in loading a drug into a membrane. (**a**) Drug loading by soaking, in which the membrane to be loaded is immersed for a certain period in a drug solution, usually under agitation. It is the most straightforward method, but the loading yield can be low, as drug entry into the membrane occurs by diffusion across a concentration gradient. As such, some of the drug present in the solution will not be loaded. Loading yields depend not only on drug concentration, but also on other loading conditions (such as solvent, temperature, pH, ionic strength and time), as well as on drug/membrane interactions, molecular size of the drug, surface area, porosity and pore size of the membrane. (**b**) Drug loading by impregnation, in which the drug solution is added to the surface of the membrane to be loaded, being fully absorbed. (**c**) Drug loading by addition, in which the drug is added to the formulation employed to prepare the membrane. The drug must be able to withstand the conditions employed in membrane preparation and, usually, it has to dissolve in the formulation. With this method, non-toxic materials and reagents have to be employed, since extensive drug loss would occur if the final drug-loaded membrane was washed to extract cytotoxic leachables.

There are three main approaches for loading a drug into a membrane (Figure 4), which are as follows: (i) soaking, in which the membrane to be loaded is immersed in a drug solution for a certain period of time under agitation, (ii) impregnation, in which a drug solution is added to the membrane, and (iii) addition, in which the drug is added to the formulation used to prepare the membrane. PHMB is amenable to all of these three membrane-loading approaches, due to its good hydrolytic, thermal and photo stabilities [18,19,97], solubility in water and ability to be dissolved in some organic solvents [28,91,94,104].

Contrary to all of the PRWDs for which this information was provided, which were consistently loaded by impregnation (Table 2), all of the above-mentioned approaches were employed in the preparation of PRMs (Table 3). Interestingly, impregnation was only employed to load PHMB into bacterial cellulose-based membranes, whereas soaking and addition were employed to load PHMB into a variety of membranes, bacterial cellulose-based membranes included. In the case of loading by impregnation, the concentration of the PHMB solutions mostly employed ranged between 0.1 and 1%, values that are within the range employed in the case of commercial PRWDs for which this information is available (0.1–1%; Table 2). The range mostly employed in loading by soaking also varied between 0.1 and 1%. Exceptions were observed in the case of the bacterial cellulose PRM12 membrane loaded by impregnation, which employed a 0.04% PHMB solution, and in the case of drug loading by soaking, concentrations of 2 and 5% were employed to load the gelatin-based PRM15 membrane and the wool-based PRM22a/PRM22b membranes, respectively. In the case of the PRM15 membranes, lower concentrations of 0.1 and 0.5% were evaluated, but much less PHMB was released and the release duration was lower than when loaded with 1 and 2% PHMB. In the case of PRM22a/PRM22b membranes, the authors have not provided drug-release curves when loaded with PHMB concentrations below 5%, although these membranes exhibited high antibacterial activity for the PHMB concentrations of 0.5% (PRM22a) and 1% (PRM22b). At this stage, it is not possible to establish any correlations between the matrix characteristics and loading yield by impregnation or soaking because few studies have reported loading yields.

Loading by addition was employed with PRMs prepared by electrospinning and by phase inversion. A comparison of the employed PHMB concentrations is not possible since, in these studies, the PHMB concentrations in the solutions employed in membrane preparation were expressed using different concentration units that cannot be interconverted (Table 3), which are as follows: (i) wt% in relation to the mass of solution, (ii) wt% in relation to the mass of polymers in the solution, and (iii) % in relation to the volume of the solution (% *w*/*v*). In the cases that employed woven membranes in which PHMB was not loaded by soaking, PHMB was added to a solution that was applied as a coating on the membrane, by either spray or dip coating (membrane immersed in a drug-containing solution for a short period). An additional approach was the addition of PHMB at concentrations ranging from 0.2 to 0.4 wt% to the growth medium employed in the production of a bacterial cellulose membrane by *Gluconacetobacter xylinus* (*G. xylinus*; PRM4), which resulted in a bacterial cellulose/PHMB composite. 

#### 5.1.3. Sterilization

Commercial WDs are either manufactured from sterile raw materials in a sterile environment (aseptic processing), as in the case of, for instance, Suprasorb^®^ C, or are sterilized in the last step in their manufacture (terminal sterilization) by gamma radiation (e.g., PuraPly^®^ AM), ethylene oxide (e.g., Telfa™ AMD), steam (autoclaving) or dry heat (e.g., Excilon^TM^ AMD), among other possible sterilization methods. Regarding the PRMs, none of the membranes were prepared using aseptic processing and only two (PRM8 and PRM16) underwent terminal sterilization (Table 3). Sterilization was also employed in the case of PRM17, but occurred just after the preparation of the membrane, i.e., before drug loading. In all other cases, only those samples used in biological testing were sterilized. It must be noted that sterilization may alter the characteristics of polymers and/or drugs [113] and, ultimately, affect drug release kinetics [114]. In fact, autoclaving altered the mechanical properties of the above-mentioned PRM17 membrane, possibly due to the crosslinking/densification of the material [107]. Regarding PHMB, it was reported that it can withstand autoclaving when in aqueous solution [97], in accordance with its good thermal and hydrolytic stability [18,19]. However, the effects of sterilization on PHMB when loaded into membranes have not been investigated. In conclusion, drug-release results obtained with PRMs that were not sterilized before the drug-release study, or were sterilized before drug loading, must be analyzed with care. In future studies, it would be important to compare the effects of the different types of sterilization on the properties relevant to the context of medicated WDs, including PHMB release kinetics.

### 5.2. Characterization

The extent of the characterization of the PRMs varied considerably, as can be observed in Table 4. As will be discussed in the following sections, the tests employed in this characterization also varied considerably. Due to these variations, direct comparisons between the different PRMs, namely in terms of their potential for AMDs, would necessarily be flawed and were not attempted.

#### 5.2.1. Physical Properties

As can be observed in Table 4, more than half of the PRMs did not undergo a physical characterization relevant to their application as WDs. For most of the other PMRs, this characterization fell short of what might be regarded as an essential physical characterization of WDs [115]. It must be noted that international standards for the physical characterization of AMDs are lacking. However, the European Committee for Standardization (CEN) developed a European Standard (EN 13726) for the physical characterization of primary WDs, i.e., dressings that are in direct contact with the wound bed, as opposed to secondary WDs, which are employed to secure in place or to absorb leakage from primary WDs. Although not specifically developed for AMDs, the different parts of this standard (Table 5) can be used for guidance. In fact, both the SMTL (Surgical Materials Testing Laboratory, Bridgend, UK) and MET (Medical Engineering Technologies, Ltd., Dover, UK), two European laboratories reputed for WD performance testing, follow most parts of this standard [116,117,118]. The International Organization for Standardization (ISO) has not developed a standard for any WDs, despite having included WDs in general in the ICS (International Classification of Standards) technical standards classification system (ICS code 11.120.20 “Wound dressings and compresses”).

**Figure 5:**
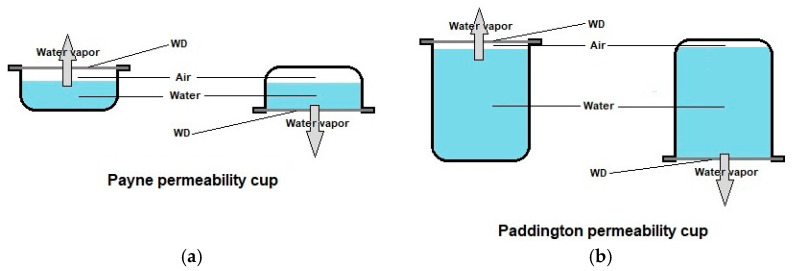
Schematic representation of the permeability cells specified for the measurement of moisture vapor transmission rate (MVTR) by standards (**a**) ASTM E96-90 (Payne permeability cup) and (**b**) EN 13726:2 (Paddington permeability cup). Both cups are depicted in the upright (left) and inverted (right) orientations. In the ASTM E96-90 standard, water evaporation through a test membrane is measured gravimetrically, by employing a Payne permeability cup located in a chamber at 23 °C or 32.2 °C and at 50 ± 2% relative humidity (RH). The Payne permeability cup contains water and its mouth is sealed with the test membrane, leaving a 19 ± 6 mm gap between the water surface and membrane. When the Payne permeability cup is placed in an upright orientation, this assay is designated “Procedure B—Water Method” or “Procedure D—Water Method”, depending on whether it is carried out at 23 or 32.2 °C, respectively. When in the inverted orientation, it is designated “Procedure BW—Inverted Water Method” and is carried out at 23 °C only. This assay can also be similarly carried out, but anhydrous CaCl_2_ is used as a desiccant inside the upright Payne permeability cup, instead of water, leaving a 6 mm gap between the sample and desiccant. In this case, the assays are designated Procedures A, C or E—Desiccant Method, depending on the temperature at which it is carried out (23 °C, 32.2 °C or 37.8 °C, respectively). In the EN 13726-2 standard, the assay is similar to the ASTM assay, with the main differences lying in the use of a larger permeability cell (Paddington permeability cup), a smaller air gap between the water surface and sample (5 mm), a different temperature (37 °C) and RH (<20%), the use of samples preconditioned at 20 ± 2 °C and 60 ± 15% RH and the absence of a method that uses a desiccant.

Of the physical properties covered by the various parts of EN 13726, only absorbency (i.e., the capacity to absorb liquid) and MVTR (moisture vapor transmission rate) were included in the characterization of at least some of the PRMs (Table 6). It must be added that absorbency was tested without strict adherence to the standard’s specifications and MVTR was not tested according to this standard, but rather according to the ASTM E96-90 standard, an American Society for Testing and Materials (ASTM) standard developed for measuring water vapor transmission of materials in general. Once again, testing did not adhere strictly to the standard’s specifications. The EN and ASTM standards differ in several specifications, namely in terms of the permeability cells employed (Figure 5). In both standards, the permeability cells can be used in the upright and inverted orientations. One important difference between these two variations is that the sample is in contact with water vapor when the cup is in the upright orientation, whereas it is in contact with water when the cup is in the inverted orientation. Thus, the latter variant might be regarded as more physiologically relevant, as WDs are in direct contact with the wound exudate. Importantly, when a set of commercial WDs were assayed according to the EN 13726 standard, statistically significant differences were observed between the values obtained with these two variants for some of the tested WDs [125].

In EN 13726-1:2002, absorptive capacity is expressed as the average mass of test solution A (a NaCl/CaCl_2_ solution that contains 42 mM Na^+^ and 2.5 mM Ca^2+^) retained per 100 cm^2^ of sample or, in the case of wound cavity-filling WDs, per gram of sample. This standard also specifies the conditions for preconditioning the samples (21 °C and 60 ± 15% relative humidity (RH)) before the assay, the temperature of test solution A during immersion (37 °C) and the duration of the immersion (30 min) [119]. In the case of PRMs (Table 6), solution A was never employed, being replaced, in most cases, by phosphate-buffered saline (PBS), and immersion lasted until equilibrium was attained, rather than for 30 min only. Absorptive capacity was also expressed in different forms, mostly as the percent mass increase in relation to dry mass, but also, in one instance (PRM17), as the percent mass increase in relation to swollen mass and, in another instance (PRM1), as the swollen to dry mass ratio. Moreover, different terms were employed to designate the same form of expressing absorptive capacity. To facilitate comparisons, the terms water uptake capacity, equilibrium water content and swelling ratio were used throughout the text and in Table 7 whenever absorptive capacity was expressed as the percent mass increase in relation to dry mass, percent mass increase in relation to swollen mass and swollen to dry mass ratio, respectively. Values of water uptake capacity in PBS varied between 233%, for the crosslinked gelatin-based PRM15 membrane, and 2000%, for the PVA/chitosan-based PRM17 membrane prepared by freeze–thaw cycling and freeze-drying. Straightforward comparisons can only be made between PRMs tested by the same group that employed the same test conditions, i.e., between PRM2a/PRM2b, and between PRM23a/PRM23b/PRM23c. PRM2a and PRM2b were both prepared by electrospinning of the same polymers, but two different approaches were employed (Table 3). PRM2a was prepared by electrospinning of a poly(esther urethane)/CA/PHMB solution (PEsUR/CA/PHMB), which resulted in a membrane composed of fibers with uniform diameters. PRM2b was prepared by co-electrospinning of PEsUR/PHMB and CA/PHMB solutions, which resulted in a mixture of fine and thick fibers. The higher absorptive capacity of PRM2b was attributed to a higher surface area, due to the heterogeneity in the fiber diameters [91]. Regarding the set of PRM23 membranes, their absorptive capacities were similar, suggesting that absorbency of bacterial cellulose was not significantly affected by the added polysaccharides (alginate, pectin or both). It is of note that sterilization by autoclaving decreased the absorbency of PRM17, which was attributed to an accompanying decrease in pore size and porosity [107]. The range of absorptive capacities of the PRMs assessed was comparable to the range of known values of a set of eleven commercial WDs (205–1766% [126]). Once again, the results must be interpreted with care, because all but one of these commercial WDs were non-medicated WDs and also because the experimental conditions employed in the determination of this parameter varied. MVTR values were only determined in three PRMs. The electrospun PEsUR/CA-based PRM2a and PRM2b were evaluated according to the ASTM E96-90 standard “Procedure D—Water Method” (Figure 5a), although different relative humidity (43%) was employed, without explicit mention of the temperature. Despite their different morphologies, fiber diameters and porosities, a similar MVTR value of ca. 204 g/m^2^/24 h was obtained for these two PRMs, which is comparable to the lower MVTR values obtained for a set of commercial WDs studied under this standard, which ranged from 50 to 9360g/m^2^/24 h [127,128]. With the bacterial cellulose-based PRM13 membrane, a much higher MVTR value of 2500 g/m^2^/24 h was obtained. However, a different assay method was employed, still in accordance with the ASTM standard. In this method, the designated “Procedure A—Desiccant Method” (Figure 5a), anhydrous calcium chloride located inside the Payne permeability cup was employed as the driving force for water vapor transmission across the test membrane [129]. As such, this assay method is not physiologically relevant.

Air and oxygen permeabilities were also determined for three PRMs. Absorbency, MVTR and air permeability can all affect the PRMs’ ability to control exudate levels. To prevent excessive exudate in the wound bed, which can result in tissue maceration [130], WDs must be able to both absorb exudate and allow its evaporation through the exchange of water vapor with the ambient air, to avoid saturation of the WDs matrices [116]. On the other hand, very high evaporation rates should be avoided, as they may cause wound dehydration and delay healing. Oxygen and air permeabilities determine oxygen levels in wounds, ultimately affecting cell and bacterial proliferation. While low oxygen levels stimulate angiogenesis [131], a high oxygen permeability might be desirable [132,133], as high oxygen levels may favor epithelialization and reduce proliferation of aerobic bacteria [134,135]. On the other hand, increased gas permeability may decrease the resistance of the WD’s outer layer to water penetration [136].

For the bacterial cellulose-based PRM13, an oxygen transmission rate of 48 cm^3^/m^2^/12 h (or 0.001 cm^3^/cm^2^/s) was obtained following the Chinese GB/T 19789-2005 standard [137], at 37 °C and 0% RH. This standard is similar to the ISO 15105-2:2003 standard [138], which was developed for measuring gas transmission rates of plastics in film form. Values of oxygen transmission rates of commercial WDs measured by employing either of these standards could not be found in the literature. In the case of the PRM2a/PRM2b membranes, air permeability was measured according to the ASTM D737-04 standard [139], which was developed for measuring air permeability of textile fabrics. PRM2a had a higher air permeability than PRM2b (2.5–4 cm^3^/cm^2^/s and 0.5 cm^3^/cm^2^/s, respectively, at a 125 Pa pressure differential), which might be due to the already mentioned differences in morphology, fiber diameter and porosity. The values obtained in both cases were within the range of values obtained with a set of commercial WDs assayed by the same method—from below 0.02 to above 78 cm^3^/cm^2^/s, the minimum and maximum values for the employed instrument, albeit at a slightly lower (100 Pa) pressure differential [136]. As such, these PRM2a/PRM2b membranes have an air permeability that might be adequate for their application as WDs.

#### 5.2.2. Drug Release

Upon application of a medicated WD to an open wound, the bioactive agent is released to the wound exudate. Thus, results of in vitro PHMB release studies can be used to predict the validity of the PRMs. Drug release kinetics are affected by characteristics of the release medium, such as pH [140], viscosity [141,142,143] and temperature. The pH affects the degree of ionization of functional groups in both the drug and the matrix, ultimately affecting drug solubility, matrix swelling and drug–matrix interactions. Viscosity affects mass transport and drug dissolution rates, and temperature affects both mass transport and solubility. Thus, the validity of these initial predictions can be improved using assay conditions similar to those of the in vivo wound environment. While it may not be possible to replicate the complexity and dynamics of this environment and may be difficult to replicate conditions such as fluid composition, volume and turnover rate, other conditions, such as temperature, pH and ionic strength, can usually be closely replicated. In addition, the fact that only one side of the WDs is in contact with the wound should also be taken into consideration, as drug release kinetics may depend on whether a drug is released from a single side or from both sides of a membrane [140]. It must be stressed that it may be possible to obtain a valid prediction by employing assay conditions that differ significantly from the corresponding in vivo conditions, providing that a mathematical correlation between PHMB-release in vitro and antibacterial action and/or wound-healing capacity (i.e., an in vivo–in vitro correlation or IVIVC) is established. The U.S. Food and Drug Administration (FDA) has issued a guidance document for industry devoted to the development, evaluation and application of IVIVC for extended-release oral dosage forms [144], but no equivalent guidelines are available for other types of extended-release dosage forms, including AMDs or other types of medicated WDs [145,146].

The exact composition of exudate varies with wound origin, type, size and location, as well as with the phase of the wound-healing process [147]. It is mostly similar to that of blood plasma [148], being rich in proteins and also containing electrolytes, saccharides, lipids, inflammatory mediators, inflammatory cells (such as lymphocytes, macrophages and polymorphonuclear leukocytes) and platelets. It also contains microorganisms, even in non-infected wounds [147,149]. pH values in the range 5–10 have been reported [150,151] for wounds, varying with wound type, wound-healing phase and presence or absence of infection (natural skin is acidic, becoming alkaline upon infection [152]) and reported temperatures lie in the 31–35 °C range [153,154,155,156,157]. In terms of viscosity, exudate can be a “viscous, sticky” fluid in non-healing wounds [158]. In the literature, various exudate models have been reported and the need for standardization of clinically relevant models has been recognized [148]. As can be observed in Table 7, various release media were employed as exudate models in the in vitro drug-release studies of PRMs, which were as follows:

(i) Distilled water;
(ii) A salt solution composed of NaCl 8.6 g/L, KCl 0.3 g/L and CaCl_2_·2H_2_O 0.33 g/L;
(iii) A 50 mM tris(hydroxymethyl)aminomethane (TRIS) solution at pH 7.4;
(iv) PBS. The exact composition varies slightly according to the laboratory; a common composition is 140 mM NaCl, 10 mM phosphate buffer, and 3 mM KCl, with a pH of 7.4 at 25 °C [159];
(v) “Simulated body fluid”, whose composition was not provided.

Water can hardly classify as an exudate model, as it differs radically from exudate, namely in terms of electrolyte composition, ionic strength, osmolality, pH and buffering capacity, to name a few parameters. The salt solution contained the major ions present in exudate, but has no buffering capacity. In turn, the TRIS solution was buffered by a non-biological molecule, but lacked the major ions present in exudate. PBS, which was employed in half of the drug-release studies with PRMs, had ion concentrations, osmolarities and pH values (not always explicitly stated) within the ranges reported for exudate. However, PBS differs significantly from exudate, namely in terms of viscosity, which is much lower (as was the case for water and the other two solutions). The composition of the “simulated body fluid” was not detailed. More complex exudate models that contain proteins, lipids and saccharides besides salts were proposed [109,160,161,162,163]. Some of these models were employed in the characterization of the antimicrobial activity of PRMs and of commercial AMDs (Section 5.2.3). However, it must be acknowledged that it might prove challenging to use these more physiologically relevant exudate models in drug-release assays, since some of their components might interfere with the assays’ readouts, requiring more complex quantification methods.

In most studies with PRMs (Table 7), the pH of the drug-release medium was not explicitly stated. All reported values (most often 7.4) were within the range of published values for wounds. Concerning temperature, the most common reported value was 37 °C. This temperature is higher than the above-mentioned range of wound temperatures, which might translate into faster drug release than in the in vivo situations.

The main driving force for drug release in non-erodible, drug-loaded membranes, such as those employed as medicated WDs, is a drug concentration gradient between the drug-containing matrix and the release medium, which is affected by the swelling and relaxation of the membrane matrix. The maximum drug-release rate will occur under conditions in which the accumulation of the drug in the release medium is considered negligible and, as such, it will not affect the dissolution of further drug molecules that are released. These conditions are designated infinite sink conditions (or just sink conditions). In pharmaceutical science studies, it is considered that infinite sink conditions normally occur when the volume of the release medium is, at least, 3 to 10 times the saturation volume for the drug under study [164]. When these conditions are not attained, finite sink conditions (also designated non-sink conditions) will prevail, and drug release will be slowed down by the drug accumulating in the release medium. Therefore, the presence or absence of sink conditions has a large effect on the drug release kinetics. In general, drug-release studies tend to be performed under sink conditions, mainly for the reason that these conditions can be easily established and checked, and because they allow a comparison of results from different studies [165]. However, sink conditions may not always occur in in vivo locations where drug-release dosage forms are employed. In wounds, given the low exudate volume and its low turnover rate and, in particular, given that the drug-releasing WD also absorbs exudate, sink conditions may not occur [166]. This aspect has been very little considered in the literature of drug-releasing WDs. However, in vitro wound models of low and high levels of exudate have already been proposed, so that the effect of different levels of exudate on drug release can be taken into account [166]. When the low exudate level model was employed, drug release was considerably slowed down and extended, in comparison to the model with unlimited sample access to the release medium. None of the studies with PRMs have indicated the type of sink conditions employed in the drug-release assays (Table 7).

Fluid leaking out of blood vessels is naturally drained and recirculated. However, in wounds, additional fluid leakage and consequent exudate accumulation occur, due to the inflammatory response that takes place, which is characterized by increased capillary permeability. Even though there is no internationally accepted standard to measure exudate production, no accepted reference “normal” rate of exudate production, and as wound exudate production varies with the wound-healing phase and wound origin, type, size and location, as well as with local and systemic factors [147,148], reported values for the exudate turnover rate in different types of wounds range from 1.7 to 28.0 mL/10 cm^2^/day [148,167], taking 1.026 g/mL as the density of exudate for unit conversion purposes [168]. The SMTL WD testing laboratory mentioned in Section 5.2.1, although recognizing that heavily exuding wounds produce around 5 mL/10 cm^2^/day, and that this value can double in the presence of infection [169], employs a higher flow rate of 1 mL/h to evaluate the fluid-handling properties of 10 cm × 10 cm WD samples (equivalent to 24 mL/10 cm^2^/day), as a compromise between physiological relevance and assay duration [169], which could serve as a guidance in selecting release medium turnover rates. The drug-release studies with PRMs have not considered medium turnover. However, in drug-release studies where the release medium is sampled and replenished with new release medium, release medium renewal occurs; depending on the sample’s area and on the aliquot’s volume, it may approach the physiological range of exudate turnover with planned sampling protocols.

Many studies with PRMs did not mention the medium volume employed. Within those that mentioned it, only three studies employed low volumes between 1 and 3 mL (PRM6, PRM11a and PRM21), with the remaining ranging from 10 to 60 mL (Table 7). Additionally, nearly all the drug-release studies were carried out in the batch mode, i.e., with samples fully immersed in the release medium. As such, given that most of these volumes will be much higher than the exudate volume in contact with a WD, the drug release kinetics in vivo may be different and, as recognized by others [170], more relevant models should be employed.

**Figure 6:**
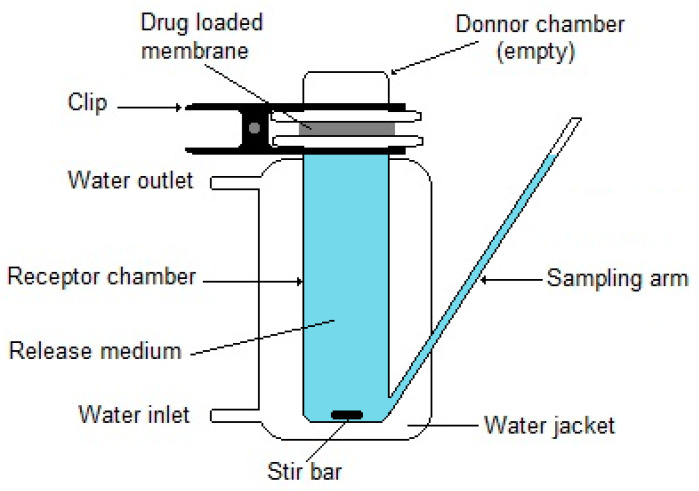
Schematic representation of a Franz diffusion cell, as employed in the study of drug release from a single face of drug-loaded membranes.

Franz diffusion cells [171] are a type of vertical diffusion cell known mostly for their use in pharmacopoeial testing of drug permeation from topical and transdermal pharmaceutical formulations [172]. They are well-suited to be used in the study of drug release from a single face of a drug-loaded membrane and have been employed in drug-release studies with membranes for WDs (e.g., [173]). In this type of application, the membrane usually employed in permeation studies as a skin model is replaced by the drug-loaded membrane, and an empty donor chamber is used (Figure 6). Although the release volumes are still larger than typical exudate volumes, the conditions of limited access to the release medium (non-sink conditions) that occur in vivo in wounds with exudate-absorbing WDs can be mimicked. For this, a low-level exudate model can be established by placing a hydrogel layer in contact with both the WD to be tested and the release medium, so that the access of the release medium to the sample is limited [166]. As mentioned earlier in this section, it may be possible to reproduce the in vivo exudate turnover rate with planned sampling protocols. Instead, flow-through Franz diffusion cells can be employed [174]. With PRMs, only a single study employed a Franz diffusion cell (PRM17, Table 7), although with unlimited access to the release medium and without mentioning its volume. Additional alternative low-volume models that might be considered are flow-through devices, such as the dynamic biofilm models employed in the antibacterial activity assays presented in Section 5.2.3 (the flatbed perfusion [175], the colony drip-flow [176] and the Duckworth biofilm [177] devices) and the “WRAP rig” [169,178], a device developed by the SMTL laboratory to evaluate the fluid-handling properties of WDs, named after the Woundcare Research for Appropriate Products project. By setting the test chamber temperature and volume to the relevant in vivo values, as well as the flow rate of an adequate exudate model, and analyzing the exudate model leaving the chamber for the released drug, the WRAP rig could also be employed in drug-release studies of drug-releasing WDs. However, none of the mentioned devices can take into account the effects of several other relevant physiological factors that affect a WD, such as sweat and skin oils, loose surface skin cells, movement, friction, pressure, shear, and varying environmental conditions. To take into account these factors, an artificial wound model placed on the skin of healthy volunteers and intermittently infused with an exudate model was proposed [160]. Although this model was developed to study the fluid-handling capacity and wear time of WDs, a modification that allows for sampling the model exudate could allow drug-release studies to be conducted. 

Most of the drug release kinetic curves obtained with PRMs showed an initial burst release, where most of the PHMB was released, followed by a plateau (Table 7). This burst release may be favorable, since it allows a powerful attack to the bacterial population thriving in the wound, but it should be followed by a sustained release. Release curves that showed a gradual, sustained release were obtained with the PRM7, PRM16 and PRM17 membranes (Table 7). These membranes were composed of polymers with different electrical charges and were prepared and loaded by employing different methods (Table 3 and Table 7), which were as follows: (i) soaking, in the case of the bacterial cellulose-based membrane PRM7 and the PVA/chitosan-based PRM17 membrane prepared by freeze–thaw cycling and freeze-drying, and (ii) addition, in the case of the PRM16 SF-based membrane prepared by freeze-drying. As such, no correlation between either polymer type, preparation method or drug-loading method and achievement of sustained drug release can be established.

Another type of release curves obtained showed two release phases (bimodal release). They were obtained with PRM8, PRM11b, PRM13 and PRM21 (Table 7). With the exception of the crosslinked PDMS-based PRM21 membrane, for which no explanation was provided by the authors and the reason for the occurrence of a bimodal release curve is not clear, the bimodal release curves of the remaining membranes could be explained. With PRM8, although an explanation was not provided by the authors, considering that this PRM was a dual drug-release system, releasing PHMB and SS (a healing-promoting polypeptide, stimulating collagen production [179]), and that SS was also a component of the membrane matrix, the bimodal release could be related to the release of SS, as when the second PHMB release started (after 24 h), 90% of SS that was released when equilibrium was attained had already been released. PRM11b is a temporarily crosslinked gelatin membrane (PRM11b), in which the crosslinked gelatin is hydrolyzed by a protease present in the membrane formulation, taking ca. 24 h for this hydrolysis to produce a noticeable effect in the drug release kinetics. Finally, the behavior of the PRM13 bacterial cellulose-based membrane loaded with PHMB/PEG was attributed to strong interactions that occur between PHMB, bacterial cellulose and PEG, holding PHMB in the early stages of the release, followed by the later expansion of the polymer network due to water penetration.

In a drug-release system, the drug-release duration, i.e., the time it takes for the cumulative released drug concentration to become constant, and the concentration of the released active drug determine the duration of the therapeutic activity of the system and, in the case of medicated WD, they may affect WD-changing frequency. Longer therapeutic activity is favorable, since it offers a lower frequency of WD change that, in turn, lowers wound exposure and favors wound stabilization, improving patient well-being and lowering costs [180,181]. To be effective, the cumulative concentration of the released drug must be within the drug’s therapeutic window, the range of drug concentrations that provide therapeutic effects with minimal adverse effects. However, as discussed earlier in this section, as the drug-release conditions in in vitro assays are very different from the in vivo conditions, drug-release duration and concentration of the released drug obtained in in vitro studies cannot be directly translated to in vivo duration/concentration.

PHMB release durations of PRMs varied from a minimum of 0.5 h to a maximum of 20 days, with the most common duration in the range of 1–24 h (Table 7). As different conditions were employed in the drug-release studies and as it was not mentioned whether sink conditions were present, it is not possible to compare the drug-release durations. The highest release durations were obtained with PRMs prepared and loaded by the following different methods: a wool-based membrane loaded by soaking with nanoliposome-encapsulated PHMB (5 days; PRM22b), an electrospun poly(ether urethane)-based membrane loaded by addition (5 days; PRM14) and a membrane prepared by freeze-drying of SF/PHMB solutions (20 days; PRM16). When the same PRM was loaded with different PHMB concentrations, increased PHMB concentrations in the soaking solution or those added to the formulation resulted in increased drug-release durations in some PRMs (PRM10, PRM13, PRM16), but not in others (PRM14 and PRM21).

Concerning the concentration of released PHMB and the maximum released concentration ([PHMB]_max_, the cumulative concentration of the drug released when equilibrium between the drug inside the membrane and the outside milieu was attained, corresponding to the plateau of the drug-release curve), as the release medium volumes employed were much larger than the exudate volumes (as observed in Section 5.2.2), these concentrations cannot be used to assess the validity of the PRMs through a comparison with the MIC for a particular bacterial species or with the PHMB therapeutic window. For studies that have indicated the concentration of released PHMB, no correlation can be established between the PHMB concentration employed in loading and the maximum released PHMB concentration since, as mentioned in Section 5.1.2, different modes of expressing the PHMB concentrations employed in drug loading were used. When the same PRM was loaded with different PHMB concentrations, an increase in the PHMB concentration added to the formulation did not always result in an increase in [PHMB]_max_; although this was true for PRM10, it was not true for the highest PHMB concentrations employed in loading PRM14 and PRM21 (Table 7). In the case of PRM14, it cannot be attributed to different loading yields, since an increase in the PHMB concentration added to the formulation always resulted in increased PHMB loading (in the case of PRM21, loading yields were not determined). Thus, no correlations could be established between the drug-release duration or [PHMB]_max_ and membrane preparation or drug-loading method, or PHMB-loading yield.

#### 5.2.3. Biological Evaluation: Antimicrobial Activity

A large number of bacterial species can be found in infected wounds, most frequently *S. aureus*, *P. aeruginosa, Proteus mirabilis* (*P. mirabilis*) and *E. coli* [182,183]. Polymicrobial infections are common, with *S. aureus* and *P. aeruginosa* as the most common association [182]. Fungi also colonize wounds, in particular *Candida albicans* (*C. albicans*) [182,184]. With the exception of PMR25b and PRM23c, whose characterization was discontinued for exhibiting lower mechanical properties and absorptive capacity than the similar PRM23a, and of PRM4, all the other 25 PRMs were evaluated for antimicrobial activity, i.e., their ability to inhibit the growth or cause a reduction in the number of viable bacteria and/or yeast cells (Table 8). Most of these studies employed, at least, one Gram-negative and one Gram-positive bacterial species, although a few studies employed only one bacterial species. *E. coli* and *P. aeruginosa* were the most employed bacteria in the Gram-negative group, while in the Gram-positive group, *S. aureus* was the most employed. *P. mirabilis*, which is also commonly found in infected wounds, was never employed.

Several types of assays have been developed to evaluate or screen the in vitro antimicrobial activity of a substance [185]. The approaches used to assess antimicrobial activity differed significantly across the various studies and a detailed presentation of these approaches is out of the scope of the present review. It must be noted that, at the time these evaluations were performed, no ISO, EN or ASTM standards for evaluating the antimicrobial activity of AMDs had been issued, although standards for the evaluation of the antimicrobial activity of other types of products, such as textiles, existed (e.g., ASTM E2922-15 [186]) and were employed in some studies. Since then, more specifically in July 2022, an EN standard (EN 17854:2022) was issued [187]. In this EN 17854:2022 standard, antimicrobial activity is assayed by inoculating calculated volumes of test suspensions of the Gram-negative bacteria *P. aeruginosa*, the Gram-positive *S. aureus* and the yeast *C. albicans* into separate samples of the AMD under evaluation. These test suspensions are prepared in a solution that contains heat-inactivated fetal calf serum (FCS), peptone and NaCl, used to simulate wound exudate. After a contact time of 24 h at 30–34 °C, the antimicrobial activity is stopped by the addition of a neutralizing solution and the number of bacteria or yeast recovered from the AMD is determined and compared to that of a negative control.

To evaluate the antimicrobial activity of PRMs, the most commonly employed assays were the agar disc diffusion assay and the inoculation assay (Table 8). In the agar disc diffusion assay, the PRMs were placed directly on agar plates onto which the bacterial strain was seeded, followed by incubation overnight or for 24 h at 37 °C. Long incubation times of 7 and 9 days, with daily changes of the agar plate, were also employed. When antibacterial activity is present, clear areas appear around the samples. These areas, which are designated inhibition zones, are areas in which bacteria do not grow; the bacterial susceptibility to the antibacterial agent is proportional to the diameter of the inhibition zone. In the inoculation assays, the PRMs or PRMs extracts were inoculated with the test bacteria, followed by bacterial quantification after incubation at 37 °C for 1 to 48 h. Bacterial quantification was carried out through optical density (OD) measurements at 620 or 650 nm, nephelometry, colony counting after spreading on agar plates, determination of viable bacterial cells by employing redox dyes, or employing microscopy. 

It is not possible to establish a correlation between antibacterial activity and the amount of PHMB loaded per unit mass of PRM samples, since very few studies determined the drug-loading yield (Table 7). Additionally, to relate the PHMB concentrations employed in PRM preparation to the presence or absence of antibacterial activity, care has to be taken since, in loading by soaking and impregnation, the concentrations refer to the concentration of PHMB in the soaking solution, while in loading by addition, they refer to the concentration in the solution employed to prepare the membrane (Table 7 and Table 8). Nearly all PRMs whose antibacterial activity was assayed were active against the tested bacterial species (Table 8). The exceptions were (i) the PRM8 membrane loaded by soaking in a 0.2% PHMB solution and the PRM10 membrane loaded by addition of 10 wt% PHMB to the formulation employed to prepare the membrane that was not active against *P. aeruginosa*, although active against other bacteria; (ii) the PRM20 membrane loaded by impregnation with 0.1% PHMB that, although active against several bacterial species and strains in a biofilm assay, did not show activity against a particular *Staphylococcus epidermidis* (*S. epidermidis*) strain, and (iii) the PRM21 membrane loaded by addition of PHMB in the range 0.1–0.5 wt%, for which no activity was detected against *P. aeruginosa*, *Acinetobacter baumannii* (*A. baumannii*) or against *Bacillus subtilis* (*B. subtilis*) biofilms. In these cases, *P. aeruginosa* was the bacterial species that most often resisted antibacterial activity. This bacterial species is known for its remarkable resistance to antibiotics [188].

In loading by soaking or impregnation, the lowest PHMB concentration that resulted in PRMs with antibacterial activity was 0.04% (PRM12, PRM18 and PRM19 membranes). Most of the remaining active PRMs were loaded with PHMB concentrations in the range 0.1–1% (Table 8). In loading by addition, as discussed in Section 5.1.2, the PHMB concentration was expressed in different modes. For the most used mode, the minimum PHMB concentration added to the formulation for which antibacterial activity was observed was 0.02% *w*/*v* (PRM5), with the remaining active PRMs loaded by adding PHMB concentrations in the range 0.04–0.4% *w*/*v*. When wt% was used, the minimum PHMB concentration that resulted in antibacterial activity was 0.1 wt% for the PRM21 membrane. However, for the PRM10 membrane, antibacterial activity was only observed for a PHMB concentration of 10 wt%. This PRM corresponds to a membrane composed of chitosan and PLA. As carboxylic groups in PLA have a p*K*_a_ value below 7, PLA will be negatively charged at physiological pH and the positively charged PHMB (and chitosan) will interact and bind to PLA. As such, this PRM may require loading with higher PHMB concentrations to exhibit antibacterial activity. However, other PRMs that contain negatively charged polymers, such as PRM5 (PLA) or PRM18/PRM19 (alginate), did not require high PHMB concentrations, suggesting that the presence of negatively charged polymers in the membrane may not be the only factor that justifies the use of high PHMB concentrations.

Most antibacterial assays were short-term assays, with 24 h being the most employed contact time. Two studies employed long contact times of 7 and 9 days, with daily renewal of the agar plate with the test bacteria, which will simulate exudate turnover in the wound. In one of these long-term studies, the antibacterial activity of PRM9 lasted for just 1 day, although it was evaluated for 7 days. In the other long-term study, it was possible to achieve long-term antibacterial activity; for the lowest PHMB concentration employed (0.1%), antibacterial activity against *S. aureus*, *A. baumannii*, and *Klebsiella pneumoniae* (*K. pneumoniae*) lasted for 3–4 days, while for the two highest PHMB concentrations (1 and 2%), the activity lasted for 7–8 days.

In a few cases where the activity of PRMs was compared to that of commercial AMDs (Table 8), in general, they possessed comparable or superior antibacterial activity. The antibacterial activity of PRM6 was (i) superior to that of a CHX-based AMD (Bactigras^®^) against Gram-positive and Gram-negative bacteria, (ii) comparable to that of a silver-based AMD (Acticoat^®^) for Gram-negative bacteria, but inferior against *P. aeruginosa*, and (iii) comparable to that of a commercial PRWD loaded by impregnation with the same PHMB concentration (Suprasorb^®^ X + PHMB) for Gram-positive bacteria, with the exception of *B. subtilis*, to which it was inferior, and for Gram-negative bacteria, with the exception of *P. aeruginosa*, to which it was also inferior. PRM12 also demonstrated antibacterial activity comparable to that of Suprasorb^®^ X + PHMB that contained a PHMB concentration 3- or 7.5-times higher, and PRM14 was superior to a commercial silver-based WD (Actisorb^®^ Silver 220), when loaded with PHMB concentrations in the range of 5–35 wt%. In a large-scale study that employed a large number of bacterial species and strains, PRM20 loaded with 0.1% PHMB exhibited antibacterial activity that was superior to a commercial silver-based AMD (Aquacel^®^ Ag), but was slightly inferior to equivalent membranes loaded with the antiseptics octinidine (OCT) and PVP-I, although these were loaded with much higher antiseptic concentrations (7.5 and 0.5%, respectively).

A different type of study looked at the storage stability of the antibacterial activity, which is an important aspect when developing AMDs for commercial use. In the only study of this type, the PRM8 membrane (Table 8) maintained its antibacterial activity against MRSA after storage for up to 6 months at 30 °C, but lost its antibacterial activity against *E. coli*, *P. aeruginosa*, *B. subtilis* and *S. aureus* for storage periods in the range of 1–6 months. For *A. baumannii*, although it was not active after storage for 1, 2 and 3 months, it was active after 6 months of storage. A commercial AMD that was assayed in parallel maintained its antibacterial activity for up to 6 months of storage.

Although wounds can be colonized by both bacteria and fungi, which both contribute to delayed wound healing [184], the antifungal activity was only evaluated in three PRMs. The PRM20 membrane reduced the biofilm of *C. albicans*, although weakly, and its antifungal activity depended on the culture medium employed, being higher in an exudate model. The growth of the same fungus was also inhibited by the PRM21 membrane. The remaining study was concerned about fungal growth during the storage of the PRM13 membrane in a humid environment. No fungal growth was observed for 6 weeks, in contrast to the equivalent samples not loaded with PHMB.

Almost all of the reported in vitro antibacterial activity assays with PRMs employed single bacterial cultures in planktonic form or as colonies on agar (Table 8). As in chronic wounds, bacteria are present as mixed-species biofilms [189] and as PHMB is less effective when bacteria are present in biofilm form [49], these are not the most relevant types of bacterial cultures to model bacteria in chronic wounds. Additionally, the effect of exudate has to be considered, since commercial AMDs, including PRWDs, have shown reduced antimicrobial activity when evaluated in vitro in the presence of human wound exudate [87]. In the evaluation of antibacterial activity of PRMs (Table 8), four studies employed bacterial biofilms, although they were static biofilm models, without growth medium renewal. PRM11a and PRM11b were able to reduce the number of viable bacteria in single-species biofilms of *P. aeruginosa* and *S. aureus*, being less active against *S. aureus*. For mixed-species biofilms, PRM11b was able to completely eradicate the biofilm, but only after 48 h of contact. In a large-scale study that employed the PRM20 membrane, antimicrobial activity with partial eradication of the biofilm was observed after 24 h for the tested bacterial strains, with the exception of a single strain of *E. epidermidis* grown in TSB (tryptic soy broth). The antimicrobial activity depended on the medium employed, being higher in the artificial exudate medium employed than in TSB, highlighting the importance of the wound exudate model selected. This PRM exhibited better performance than a silver-based commercial AMD (Aquacel^®^ Ag), but slightly inferior performance than the equivalent membranes loaded with OCT and PVP-I, although containing much higher concentrations.

**Figure 7:**
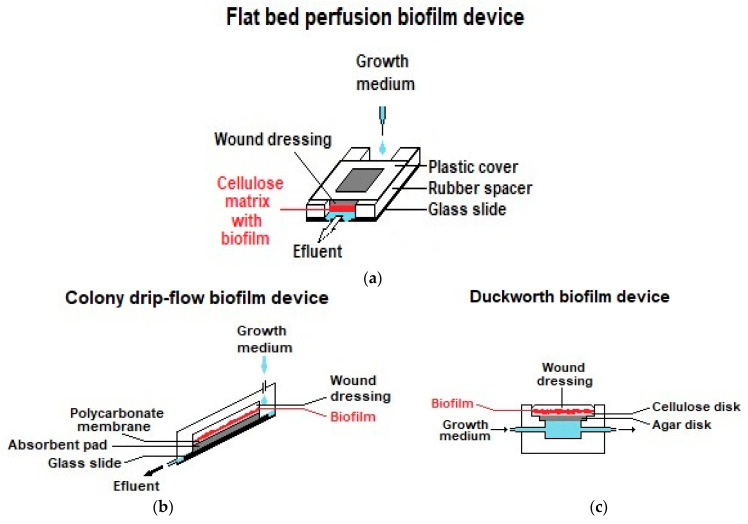
Schematic representation of in vitro dynamic biofilm models employed in the evaluation of the antibacterial activity of AMDs. (**a**) The flatbed perfusion biofilm model is composed of a cellulose matrix that contains a biofilm held on an inclined glass slide. Growth media are perfused through the cellulose matrix and collected for bacterial quantification. (**b**) The colony drip-flow biofilm model is composed of a semipermeable polycarbonate membrane in which biofilms are grown, which is placed on an absorbent pad sitting on an inclined glass slide. Growth medium is dripped on the microscope slide, perfusing the absorbent pad. (**c**) The Duckworth biofilm device is a flow device that contains wells with an agar disk fed with flowing growth medium from beneath, on top of which semipermeable cellulose-based disks can be placed and inoculated for biofilm growth. AMDs are assayed by direct application on the biofilm.

Dynamic biofilm models have been developed for the study of the antibiofilm activity of antimicrobial substances. Although not employed to study PRMs, this type of model has already been employed to evaluate the antibacterial activity of commercial AMDs. In an evaluation of Inadine^®^, a PVP-I-based AMD, and of Acticoat^®^, a silver-based AMD, a flatbed perfusion biofilm device (Figure 7a) that contained single-species biofilms of *S. aureus* or *P. aeruginosa* and FCS as an exudate model was employed [175]. Inadine^®^ AMD showed an initial strong antibacterial effect that faded away with time, allowing bacterial population recovery, while ActiCoat^®^ showed a gradual antibacterial effect, lasting more than 24 h. A commercial PRWD (Excilon^TM^ AMD) was studied in the colony drip-flow biofilm model (Figure 7b) with biofilms of MRSA or *P. aeruginosa*, employing TSB as the exudate model, and was compared to a silver-based AMD (Silvercel^®^) and to WDs without antibacterial agents [176]. After treatment for 72 h, no AMD was able to completely eradicate the bacterial population, to prevent biofilm formation or even decrease the initial bacterial population. However, the bacterial load was significantly lower than that recorded for the treatment with the WDs without antibacterial agents. The Duckworth biofilm device (Figure 7c) was employed in an evaluation of undisclosed commercial AMDs, with a mixed biofilm model of *P. aeruginosa* and *S. aureus* [190]. The AMDs were studied after 24, 48 and 72 h of contact with established biofilms, at 33 °C, employing an undisclosed growth medium. A negligible reduction in bacterial counts was obtained.

Several in vivo infected wound models have been proposed for the study of antimicrobial compounds [191], which are mostly in vivo wound-healing models (presented in Section 5.2.5) colonized with bacteria. The evaluation of the antibacterial activity occurs by bacterial quantification and histology. In the evaluation of PRMs, two studies employed in vivo wound models (Table 8). In the first study, in which PRM1 was evaluated with a rat full-thickness excisional wound model (Section 5.2.5) infected with *P. aeruginosa* [90], surface antibacterial activity could be detected after 8 days, reaching its maximum after 12 days. The lack of antibacterial activity in the early stage was attributed to the water provided to the wound by the hydrated PNIPAAm-based hydrogel employed that favored bacterial growth [90]. In the case of deep tissue infection, no significant difference in bacterial counts occurred when compared to the negative control. This was expected, given that PHMB has poor skin penetration [28]. In another rat infected full-thickness excisional wound model study, *A. baumannii* was employed to evaluate the PRM15 membrane. When this PRM was assayed in vitro (Table 8), it showed antibacterial activity against both Gram-negative and Gram-positive bacteria that lasted for at least 7 days. In the in vivo assay, a decrease in infection was observed, in particular in the early stage of treatment, probably due to the higher concentration of released PHMB. After 21 days, bacteria were still present, although in significantly lower numbers than in the case of the control group (unloaded membrane), and some animals still showed infection symptoms.

To conclude, there is a need for evaluating the in vitro antibacterial activity of PRMs that employ dynamic, multi-species biofilm models, since antibacterial activities obtained with AMDs assayed with dynamic biofilm models were lower than when assayed with static biofilm models. In in vivo evaluations that employ infected wound models, although the antibacterial activity lasted much longer than in the in vitro assay, no complete resolution of the wound infection occurred, highlighting the need for care in the interpretation of the results obtained with these two model types.

#### 5.2.4. Biological Evaluation: Biocompatibility

As is the case with all medical devices, the PRMs must be evaluated for biocompatibility, i.e., for potential biological risks that may arise from their intended use. A set of test methods for the evaluation of the biocompatibility of medical devices that are in contact with breached or compromised surfaces is provided by ISO 10993. According to part 1 of this standard (ISO 10993-1:2018) [192], AMDs intended for prolonged used (i.e., <24 h to 30 days for the sum of single, multiple or repeated durations of the contact) must address the following endpoints: cytotoxicity, irritation or intracutaneous reactivity, material-mediated pyrogenicity, acute systemic toxicity, subacute toxicity and implantation effects.

Only twelve PRMs were tested for biocompatibility (Table 9). Except for two cases, the only endpoint assessed was in vitro cytotoxicity, taken as an indirect measure of the damage that the PRMs may cause to cells present in the wound bed and its surroundings. A straightforward comparison of the results is, once again, precluded due to the significant variations in the methodologies employed, namely in terms of cell models, material tested (the membrane itself or a membrane extract), mode and duration of the contact between the cells and the endpoint/assay. ISO 10993-5:2009, which describes a scheme for assessing the cytotoxicity of medical devices [193], classifies cytotoxic materials as those that cause a reduction in the parameter assessed (e.g., number of cells, amount of protein or reduction in a vital dye) by more than 30%, after 24 h of incubation. In the case of the PRMs assayed, incubations lasted from 3 to 21 days, but mostly 24 h.

Depending on the concentration, PHMB may exhibit in vitro cytotoxicity [194]. In PHMB loading by soaking, a dependence of the result of the cytotoxicity evaluation on the conditions employed in the preparation of sample extracts was found in a study with the PRM7 membrane. In this study, the result obtained depended on the sample/liquid ratio employed in the preparation of the test extract and on the duration of the cell incubation with the extract, with higher ratios and longer incubation times resulting in cytotoxicity [97]. Improvement in cell toxicity was obtained by encapsulating PHMB at a concentration of 1 and 5% that, when not encapsulated, was cytotoxic (PRM23 and PRM22b; Table 9). PHMB concentrations of 1% employed in soaking resulted in non-cytotoxic PRMs. However, for this concentration, PRM22a was cytotoxic, although its cytotoxicity was due to the cationic nanoliposome employed.

In the case of loading by the addition of PHMB to the formulation, in which different forms of expressing the PHMB concentration in the solution were used (see Section 5.1.2), precluding a global comparison, cytotoxicity did not occur with the membranes prepared by employing formulations that contained concentrations of PHMB of 0.25% *w*/*v* in relation to the formulation volume, up to 5 wt%, when expressed in relation to the polymer mass in the formulation, and up to 0.3 wt% in relation to the solution mass (Table 9).

Among the PRMs, two studies have evaluated other categories of biological effects covered by the ISO 10993 standard, in addition to cytotoxicity (Table 10). The most complete study was carried out with PRM15. It evaluated irritation/sensitization and subcutaneous implantation effects, in addition to cytotoxicity. This PRM had favorable results, with the exception of showing minimal intradermal stimulation. The effects of subcutaneous implantation in rats and of sensitization in healthy human volunteers were also evaluated with the PRM6 membrane. Lower inflammatory responses than those with a CHX-based commercial dressing (Bactigras^®^) were observed in the subcutaneous evaluation and no sensitization was detected in the study with human volunteers. The irritation potential of the PRM17 membrane was evaluated by employing an assay that is out of the scope of the ISO 10993-23:2021 standard, dedicated to the evaluation of irritation effects of substances [195], which was the hen’s egg chorioallantoic membrane test (HET-CAM test) [196]. Upon application of PRM samples on the CAM for 5 min, no hemorrhage, coagulation or lysis occurred in the blood vessels of the CAM, indicating the absence of irritation.

Although it could be advantageous if WDs possessed hemostatic capacity, evaluation of the hemostatic capacity of PRMs was reported in a single case. The PAm-based PRM18 woven membrane coated with alginate that contained silver nanoparticles (AgNPs) and PHMB accelerated blood clotting when evaluated with a qualitative blood-clotting assay, in which fresh, non-anticoagulated human blood was applied to the membrane and clot formation was visually evaluated.

#### 5.2.5. Biological Evaluation: Wound Healing

Antimicrobial activity and absence of cytotoxicity do not necessarily equate with improved wound healing. It is also critical that PMRs do not adversely affect the healing process. Thus, wound-healing testing should be regarded as a fundamental component of the characterization of AMDs. The evaluation of wound-healing capacity is largely carried out in vivo, using animal models. The most frequently employed laboratory animals are mice, rats, pigs and rabbits [197]. Pig wound models have several major unique advantages, namely their skin that is anatomically and physiologically more similar to human skin than the skin of laboratory animals [198]. Yet, pigs are rarely used, due to their cost and difficulty of handling, to name a few factors. In vitro models have also been developed, employing cultured mammalian cells and tissues. They offer more controlled and affordable studies than in vivo models, can produce results more rapidly and involve less ethical considerations. However, they cannot reproduce the biological mechanisms involved in wound healing, restricting the evaluation to a single aspect of wound healing, i.e., cell migration [199].

**Figure 8:**
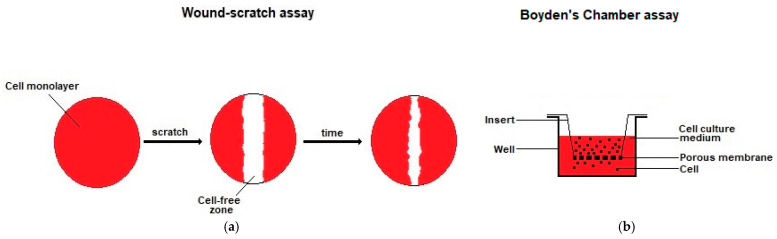
Schematic representation of the in vitro assays employed in the evaluation of the wound-healing capacity of PRM6. (**a**) Wound-scratch assay, in which a linear gap is created in a cell monolayer by scratching the monolayer with the tip of a sterile pipette tip. The WD is assayed by applying extracts in cell culture medium to the cell monolayer. (**b**) Boyden’s chamber assay (also known as transwell migration assay or chemotaxis assay), in which a cell suspension is contained in hanging inserts with a porous membrane that are immersed in cell culture medium. The WD is assayed by applying extracts in cell culture medium to wells. After incubation, cells that migrate into the well are counted.

As can be observed in Table 11, of the 28 PMRs, only 7 were evaluated for wound-healing capacity, 5 in rat models (PRM1, PRM2b, PRM6, PRM13 and PRM15) and 2 in case studies (PRM4 and PRM17). One of the PRMs evaluated in a rat model was also evaluated in an in vitro mammalian cell model (PRM6). It was evaluated in terms of cell migration, employing L929 mouse fibroblasts in both wound-scratch [200] and Boyden’s chamber [201] assays to test sample extracts in cell culture medium. In the wound-scratch assay (Figure 8a), cell migration into a linear cell-free gap in a cell monolayer was monitored by microphotography, after the addition of the sample extract to the monolayer. In the Boyden’s chamber assay (Figure 8b), cells were seeded into a cell culture hanging insert located inside a well that contained the cell culture medium. The sample extract was added to the well and the cells that migrated into the well were fixed and stained for cell counting. In both assays, cell migration in the assay of PRM6 was comparable to that of the negative control (cell culture medium) and to that of a commercial CHX-based AMD (Bactigras^®^). This result agrees with in vitro studies that show that the migration capacity of keratinocytes and fibroblasts is not affected when treated with low PHMB concentrations (0.001%) [194].

These two in vitro wound healing assays are not the most representative models of a wound. In the Boyden’s chamber assay, cell–cell and cell–matrix interactions are not reproduced, as cells are added as suspensions; in the wound-scratch assay, as it is a two-dimensional model, it does not adequately represent the complexity of a wound. Other models that more closely resemble a wound are three-dimensional (3D) models in which cell-free 3D zones are created in 3D cell cultures in collagen matrices [202]. These cell-free zones are created by punching out collagen cylinders and filling the cylindrical voids with cell-free collagen. Cell migration into these voids can be monitored by microphotography after staining. In addition to the assay of test sample extracts, WDs can be assayed in both the wound-scratch and in the 3D model by direct application to a monolayer with a scratch [203] or to a 3D collagen matrix [204].

In the in vivo wound-healing evaluation of PMRs, rats were consistently used and wounds were induced either by heat or surgically (Table 11). These types of animal wound models are classified as (i) burn wound models, in which a burn is created on the shaved skin of an animal with the aid of steam, wax or a heated metal plate, and (ii) incisional wound models, which are sutured surgical incisions, or (iii) excisional full-thickness cutaneous wound models, in which all the skin layers (epidermis, dermis and subcutaneous fat) are surgically removed, respectively [205]. The rat full-thickness excisional wound model was employed in an evaluation of wound-size evolution after application of PRMs on the wound and compared to untreated wounds, to wounds treated with an equivalent, unloaded membrane or with a commercial AMD. Wound closure was always faster for wounds treated with PRMs. It was achieved in 12 days for the PRM1 membrane, while wounds treated with the PRM6 membrane required more than 21 days to fully close, yet still performed better than a commercial CHX-releasing AMD (Bactigras^®^). With this wound-healing model, in addition to wound size or area, other parameters that were taken as indicators of wound healing were the area of the wound covered by collagen [96], the occurrence of neovascularization and the intensity of the inflammatory response [105] (Table 11). For treatment for 14 days with the already mentioned PRM6 membrane, collagen formation was higher when compared to treatment with Bactigras^®^ and the PRM15 membrane showed no effect on the neovascularization capacity or on the levels of inflammatory factors when compared to the unloaded membrane.

In the evaluation of the PRM13 membrane, by employing the rat incisional wound model, complete wound closure occurred after 28 days of treatment, in contrast to what was reported for the treatment with two commercial non-AMDs, which did not show full wound closure within this time period. Finally, the rat burn wound model was employed to study the wound-healing capacity of the PRM2b membrane. After 16 days of treatment, an almost regenerated epidermal layer was found, containing new capillaries, sebaceous glands and hair follicles. In contrast, an ongoing inflammatory response was still present in the untreated group and in the group treated with an unloaded membrane.

Concerning the reported case studies (Table 11), a dog with infected contact ulcers on its rear limbs, to which the PRM17 membrane was applied to the wound and changed daily in the first week and every other day during the following 3 weeks, resulted in a completely healed wound after that time, while a contralateral wound treated with a commercial non-medicated WD did not heal completely. In two human case studies of which very few details were provided, two diabetic toe amputation wounds healed completely after 4 and 60 days of treatment with the bacterial cellulose/PHMB composite membrane PRM4.

In summary, no PRMs tested for in vivo wound-healing capacity affected the wound-healing process, resulting in complete wound healing. They show promise for their application as AMDs.

#### 5.2.6. Clinical Trials and Patents

A search carried out on 20/11/2022 using the European [206], American [207], Japanese [208] and Chinese [209] clinical trials registries, as well as the International Clinical Trials Registry Platform [210], found eleven studies with PRWDs. These studies were started between 2005 and 2022, with four of them in the last three years. The eleven clinical trials included PRWDs with commercial tradenames that are present in Table 2 (DracoFoam, Fitostimoline^®^, Kerlix^TM^ and PuraPly^®^), a PRWD that appears to have been discontinued (COPA^®^ AMD), and three PRWDs without a tradename. Two of these clinical trials with PRWDs without a tradename—registry nos. NCT02652169 and NCT02643680 of the American clinical trials registry database [207]—employed PRWDs whose compositions are similar to those of PRM9 (a PRF-based PRM) and of PRM6 (a bacterial cellulose/SS-based PRM), respectively (Table 3). In addition, the principal investigators of these clinical trials are also co-authors of the studies with PRM6 and PRM9, suggesting that these PRMs were further developed and have reached the clinical trial stage.

A search using the Google Patents search engine [211] carried out on November 22, 2022 with the search string “wound* AND dressing* AND (“poly(hexamethylene biguanide)” OR “polyhexamethylene biguanide” OR polyhexanide OR PHMB) AND (control* OR delay* OR sustain* OR retard* OR release* OR deliver* OR elut*) after:priority:20180101” resulted in 628 published patents in the last five years, highlighting the high recent patenting activity related to wound dressings based on the release of PHMB.

## 6. Concluding Remarks

Research on PRMs for their application as AMDs has resulted in a number of membranes that were characterized to different extents. The drug-release studies carried out employed in vitro models and conditions that were far from approaching the physiological conditions and, in many cases, information concerning the conditions under which the studies were carried out was incomplete or missing. With very few exceptions, the effect of sterilization was not considered. As the type of sink conditions employed was not mentioned, as it is likely that non-sink conditions will prevail in wounds covered with a WD, and as sterilization may alter the drug release kinetics, the obtained drug-release results are unlikely to have physiological significance. Thus, a full account of the conditions employed in drug-release assays with PRMs is desired, as well as the use of in vitro models for drug-release studies that better simulate physiological conditions.

The physical characterization of the PRMs fell short of an essential physical characterization of WDs and different assays and assay conditions were employed to evaluate the same property, precluding a comparison of the results. In general, the PRMs showed antibacterial activity against the several bacterial species and strains tested, mostly employing single bacterial cultures in planktonic form, in colonies on agar or as static biofilms. Since the most representative models of the infected wound bed are multi-species biofilms under dynamic conditions, an evaluation of the antibacterial activity of the PRMs that uses this more realistic in vitro model or in vivo models is missing. In a few cases in which the antibacterial activity was compared to that of commercial PRWDs or to other types of AMDs, usually, it was comparable or superior. In general, no cytotoxicity was detected and, when detected, it occurred for PRMs that were loaded using the highest PHMB concentrations. Some PRMs were evaluated for wound-healing capacity, by mostly employing in vivo rodent wound-healing models, but also in two human and in one animal case study. All PRMs that were evaluated for wound-healing capacity resulted in successful outcomes and performed better than the selected non-medicated WDs or commercial AMDs, showing faster wound closure. As such, they show promise for further development. In fact, two AMDs that are likely to be based on two PRMs are undergoing clinical trials.

The next generation of PRMs may well include the use of PHMB in some novel types of WDs. Among them is the use of PHMB (i) associated with nanocarriers, (ii) on WDs prepared by additive manufacturing and (iii) on smart electronic WD platforms. With the exception of the recent use of PHMB encapsulated in nanoliposomes in PRM19, the use of nanocarriers in combination with PHMB remains unexplored; a small number of studies with PHMB that involve nanocarriers exist [212,213,214,215,216], but the resulting nanosystems are yet to be included in membranes. WDs prepared by 3D printing offer on-demand manufacture of WDs with shapes that conform to a particular body location in a particular patient [217,218,219]. Antibacterial agents have already been included in WDs prepared by 3D printing [219], although systems that employ PHMB have not appeared yet. Smart electronic WD platforms have appeared recently [220]. They act as WDs, containing different types of (bio)sensors for real-time monitoring of the wound and active drug-delivery systems that can deliver drugs for wound treatment as needed. Given its stability and performance, PHMB is an excellent candidate for inclusion as an antimicrobial agent in 3D-printed WDs and in the active drug-release system of smart electronic WD platforms.

## Figures and Tables

**Table 1:** Minimum inhibitory concentration (MIC) and minimum bactericidal concentration (MBC) values of polyhexanide (PHMB) against bacterial species relevant to infected wounds.

Bacteria	MIC (µg/mL)	MBC (µg/mL)	Ref.
*S. aureus*	1	2	[53]
	0.5	1	[54]
MRSA	2	2	[53]
*E. coli*	0.5	1	[54]
	2	2	[55]
*P. aeruginosa*	2	2	[54]
	8	8	[55]

*E. coli*—*Escherichia coli*; MRSA—methicillin-resistant *Staphylococcus aureus*; *P. aeruginosa*—*Pseudomonas aeruginosa*; *S. aureus*—*Staphylococcus aureus*.

**Table 2:** Commercially available PHMB-releasing wound dressings (PRWDs), in alphabetical order.

Commercial Name	Format	Material	PHMB Loading Mode/[PHMB]	Duration of Antimicrobial Activity	Sources
ActivHeal^®^ PHMB	Foam, pad	Polyurethane	Impregnation/nd	7 days	Advanced Medical Solutions, Ltd., Winsford, UK; [85]
CelluDress-PHMB	Pad	Polyester + viscose	nd ^1^	nd	Medicareplus International Ltd., Wembley, UK; [68]
Curity^TM^ AMD Antimicrobial Woven Sponges	Sponge, strip	Cotton	Impregnation/0.2%	Up to 3 days	Cardinal Health, Dublin, OH, USA; [86]
DracoFoam PHMB	Foam	Polyurethane	nd	Up to 7 days	Dr. Ausbüttel & Co. GmbH, Dortmund, Germany; [87]
Excilon^TM^ AMD	Sponge, gauze	Polyester + rayon	Impregnation/0.2%	Up to 3 days	Cardinal Health, Dublin, OH, USA; [86]
Fitostimoline^®^ Plus Gauze	Gauze	nd	nd ^2^	nd	Farmaceutici Damor S.p.A., Napoli, Italy
Gemcore360°^TM^ PHMB Foam Border Dressing	Foam	nd	Impregnation/nd	Up to 7 days	GEMCO Medical, Hudson, OH, USA; [86]
Gemcore360°^TM^ PHMB Non-Adhesive Foam Dressing					
Kendall™ AMD	Foam, disc	Polyurethane	Impregnation/0.5%	Up to 7 days	Cardinal Health, Dublin, OH, USA; [86]
Kerlix™ AMD	Gauze, sponge	Cotton	Impregnation/0.2%	Up to 3 days	
McKesson PHMB Hydrophilic Foam Dressing	Foam	Polyurethane	Impregnation/0.8–1%	Up to 7 days	McKesson Medical-Surgical, Irving, TX, USA; [86]
PuraPly^®^ AM	Sheet, disc	Crosslinked ECM	nd	nd	Organogenesis Inc., Canton, MA, USA; [88]
Sterilux^®^ AMD Antimicrobial Gauze	Gauze, sponge	Cotton	nd/0.1% PHMB (+0.02% BKC)	Up to 7 days	Hartmann USA, Inc., Rock Hill, SC, USA; [86]
Suprasorb^®^ X + PHMB	Sheet	Bacterial cellulose	nd/0.3%	nd	Lohmann & Rauscher GmbH & Co. KG, Neuwied, Germany; [89]
Suprasorb^®^ P + PHMB	Foam	Polyurethane	nd		
Telfa™ AMD	Pad, island	Cotton	Impregnation/0.2%	Up to 3 days	Cardinal Health, Dublin, OH, USA; [86]
Tielle^TM^ PHMB	Foam	Polyurethane	Impregnation/nd	nd	3M/KCI, St. Paul, MN, USA

nd–Not disclosed by the manufacturer. ^1^ Contains PHMB complexed with an undisclosed surfactant. ^2^ Contains PHMB and an aqueous extract of *Triticum vulgare*. BKC–benzalkonium chloride; ECM–extracellular matrix.

**Table 3:** Composition and methods employed in the preparation of PHMB-releasing membranes (PRMs) developed for antimicrobial dressings (AMDs).

ID	Matrix Composition	Membrane Preparation	Drug-Loading Method and Drug-Loading Yield	Ref.
PRM1	PNIPAAm-based copolymer ^1^	Free-radical polymerization.	-Soaking, overnight, RT. [PHMB]: 0.1 and 1% *w*/*v*. Agitation not mentioned. -Yield: nd.	[90]
PRM2a	PEsUR/CA	Non-weaving: electrospinning of a PEsUR/CA/PHMB solution in DMF/THF.	-Addition. [PHMB]: 1 wt% relative to polymer mass. -Yield: nd.	[91]
PRM2b		Non-weaving: co-electrospinning of CA/PHMB and PEsUR/PHMB solutions in DMF/THF.		
PRM3	Chitosan/ PEO	Non-weaving: electrospinning of a chitosan/PEO/PHMB solution in acetic acid, followed by crosslinking with glutaraldehyde in a vapor phase.	-Addition. [PHMB]: 0.15 and 0.3% *w*/*v* relative to solution volume. ^2^ -Yield: nd.	[92]
PRM4	Bacterial cellulose	Non-weaving: bacterial cellulose produced by *G. xylinus* ^3^ in PHMB-containing growth medium.	-Bacteria grown in culture medium supplemented with [PHMB] = 0.2–0.4 wt% relative to solution mass. -Yield: nd.	[93]
PRM5	PLA	Non-weaving: electrospinning of a PLA/PHMB solution in chloroform/acetone/formic acid.	-Addition. [PHMB]: 0.02–0.25% *w*/*v* relative to solution volume. -Yield: nd.	[94]
PRM6	Bacterial cellulose	Non-weaving: bacterial cellulose produced by *A. xylinum.* ^3^	-Impregnation. [PHMB]: 0.3% *w*/*v*. Additionally impregnated with SS 1%. No further conditions mentioned. -Yield: nd.	[95,96]
PRM7		Non-weaving: bacterial cellulose produced by *K. xylinus*.	-Soaking for 48 h, 20 °C, with shaking. [PHMB]: 1% *w*/*v*. -Yield: 8.9 µg/mg of sample.	[97]
PRM8	SS/PVA/Gly	Phase inversion, thermally induced phase separation: freeze-drying of a SS/PVA/Gly solution, followed by immersion in a Gly solution and drying.	-Soaking for 20 min. [PHMB]: 0.2% *w*/*v*. No further conditions mentioned. -Terminal sterilization by gamma radiation. -Yield: nd.	[98]
PRM9	PRF/silicone	Weaving or non-weaving: ^4^ silicone gauze spray-coated with a PRF/PHMB/trypsin solution.	-Addition. [PHMB]: nd. -Yield: nd.	[99]
PRM10	Chitosan/ alginate	Phase inversion: solvent evaporation of a chitosan/alginate/PHMB/Pluronic F68 solution, followed by crosslinking with CaCl_2_.	-Addition. [PHMB]: 1% and 10 wt% relative to polymer mass. -Yield: 7–73 µg PHMB/mg of sample.	[100]
PRM11a	Gelatin	Phase inversion, crosslinking: transglutaminase-induced crosslinking of gelatin solutions with PHMB and EDTA.	-Addition. [PHMB]: 0.4% *w*/*v* relative to solution volume. -Yield: nd.	[101]
PRM11b		Phase inversion, crosslinking: temporary transglutaminase-induced crosslinking of gelatin solutions that contain PHMB, EDTA and a protease.		
PRM12	Bacterial cellulose	Non-weaving: commercial bacterial cellulose WD.	-Impregnation, 2 h, RT. [PHMB]: 0.04 and 1% *w*/*v* PHMB (+0.1% UDAPB). -Yield: 0.024% (0.04% PHMB sample); 0.076% (0.1% PHMB sample).	[102]
PRM13		Non-weaving: commercial bacterial cellulose.	-Soaking for 24 h. [PHMB]: 0.1–0.5% *w*/*v* (contained PEG). No further conditions mentioned. -Yield: nd.	[103]
PRM14	PEtUR	Non-weaving: electrospinning of a solution of a commercial PEtUR in TFE that contains PHMB.	-Addition. [PHMB]: 5–35% wt% relative to polymer mass. -Yield: 0.12–0.81 mg of PHMB.	[104]
PRM15	Gelatin	Phase inversion, crosslinking: gelatin solution crosslinked with glutaraldehyde, followed by addition of Gly and solvent evaporation. Crosslinked gelatin membrane placed on a collagen layer and covered with a silicone layer.	-Soaking for 48 h. [PHMB]: 0.25–2% *w*/*v*. No further conditions mentioned. -Yield: nd.	[105]
PRM16	Reg-SF	Phase inversion, thermally induced phase separation: freeze-drying of a reg-SF/Gly/PHMB aqueous solution.	-Addition. [PHMB]: 0.5–10 wt% relative to polymer mass. -Terminal sterilization by gamma radiation. -Yield: nd.	[106]
PRM17	PVA/chitosan	Phase inversion, thermally induced phase separation: freeze-drying after freeze–thaw cycling of PVA/chitosan aqueous solutions.	-Soaking for 24 h, at 36 °C, with shaking. [PHMB]: 0.5% *w*/*v*. -Sterilization by autoclaving before drug loading. -Yield: 13–23 µg PHMB/mg of dry sample.	[107]
PRM18	PAm/alginate/ AgNP	Weaving: commercial woven PAm treated with an oxygen plasma and dip coated with an alginate solution that contains AgNPs and PHMB, followed by crosslinking with CaCl_2_.	-Addition. [PHMB]: 0.04–0.2% *w*/*v*. -Yield: nd.	[108]
PRM19	Cotton/alginate/ AgNP	Weaving or non-weaving: ^4^ commercial cotton gauze dip-coated with an alginate solution that contains AgNPs and PHMB, followed by crosslinking with CaCl_2_.		
PRM20	Bacterial cellulose	Non-weaving: bacterial cellulose produced by *A. xylinum.* ^3^	-Impregnation, overnight, 4 °C. [PHMB]: 0.1% *w*/*v* (+0.1% UDAPB). -Yield: nd.	[109]
PRM21	PDMS-based elastomer	Phase inversion, crosslinking: reaction between polysiloxane oligomers with and without vinyl groups.	-Addition. [PHMB]: 0.1–0.5 wt% relative to solution mass. -Yield: nd.	[110]
PRM22a	Wool	Weaving: commercial woven wool fabric treated with a non-ionic surfactant and a protease.	-Soaking overnight, 70 °C. [PHMB]: 0.2–5% *w*/*v*. Agitation not mentioned. -Yield: nd.	[111]
PRM22b			-Soaking overnight, 70 °C. [PHMB]: 0.2–5% *w*/*v*, encapsulated in cationic nanoliposomes. Agitation not mentioned. -Yield: nd.	
PRM23a	Bacterial cellulose/ alginate	Non-weaving: commercial bacterial cellulose/alginate/PEG solution crosslinked with CaCl_2_, followed by freeze-drying.	-Soaking, 24 h. [PHMB]: nc. No further conditions mentioned. -Yield: ca. 100% of PHMB in the soaking solution.	[112]
PRM23b	Bacterial cellulose/pectin	Non-weaving: commercial bacterial cellulose/pectin/PEG solution crosslinked with CaCl_2_, followed by freeze-drying.	-Soaking, 24 h. [PHMB]: nc. No further conditions mentioned. -Yield: 98% of PHMB in the soaking solution.	
PRM23c	Bacterial cellulose/ alginate/pectin	Non-weaving: commercial bacterial cellulose/alginate/pectin/PEG solution crosslinked with CaCl_2_, followed by freeze-drying.		

^1^ PNIPAAm/NtBAAm/A-Lys copolymer crosslinked with PEG-DA; ^2^ a weight-average MW of 2600 g/mol was employed to convert molarity into % *w*/*v* [58]; ^3^ *A. xylinum* and *G. xylinus* were renamed to *K. xylinus*; ^4^ Not clear whether a woven or non-woven gauze was employed. nc—Not clear; nd—not determined or not disclosed. AgNP—Silver nanoparticles; A-Lys—acryloyl-lysine; *A. xylinum*—*Acetobacter xylinum*; CA—cellulose acetate; DMF—dimethylformamide; EDTA—ethylenediaminetetraacetic acid; Gly—glycerol; *G. xylinus*—*Gluconacetobacter xylinus*; *K. xylinus*—*Komagataeibacter xylinus*; NtBAAm—*N*-tert-butylacrylamide; PAm—polyamide; PDMS—polydimethylsiloxane; PEG—poly(ethylene glycol); PEG-DA—poly(ethylene glycol) diacrylate; PEO—poly(ethylene oxide); PEsUR—poly(ester urethane); PEtUR—poly(ether urethane); PLA—poly(lactic acid); PNIPAAm—poly(*N*-isopropylacrylamide); PRF—platelet-rich fibrin; PVA—poly(vinyl alcohol); Reg-SF—regenerated silk fibroin; RT—room temperature; SS—silk sericin; TFE—2,2,2-trifluoroethanol; THF—tetrahydrofuran; UDAPB—undecylenamidopropyl betaine; WD—wound dressing.

**Table 4:** Overview of the studies carried out with PHMB-releasing membranes (PRMs) developed for antimicrobial dressings (AMDs). Membrane IDs and corresponding bibliographic references are the same as in Table 3.

					Biological Characterization				
	Physical Characterization						Biocompatibility		
ID	Absorptive Capacity	MVTR	Air or Oxygen Permeability	Drug Release	Antibacterial Activity	Antifungal Activity	Cytotoxi-city	Other Biological Effects	Wound Healing
PRM1				^1^					
PRM2a									
PRM2b									
PRM3									
PRM4									
PRM5									
PRM6									
PRM7									
PRM8									
PRM9									
PRM10									
PRM11a									
PRM11b									
PRM12									
PRM13						^2^			
PRM14									
PRM15				^3^					
PRM16									
PRM17									
PRM18									
PRM19									
PRM20									
PRM21									
PRM22a									
PRM22b									
PRM23a									
PRM23b									
PRM23c									

Red—Not determined; green—determined. MVTR—Moisture vapor transmission rate. ^1^ Unusual drug-release study, carried out by determination of the antibacterial activity of aliquots from a drug-release assay. ^2^ Study concerned only about fungal growth during storage. ^3^ Unusual drug-release study, carried out by quantification of residual PHMB in samples that were employed in a drug-release assay.

**Table 5:** European Standards (EN) applicable to the physical characterization of primary wound dressings.

Standard	Title	Comment	Ref.
EN 13726-1:2002	Test methods for primary wound dressings. Part 1: Aspects of absorbency	Provides information concerning the evaluation of the quantity of fluid that a wound dressing can absorb and retain when compressed.	[119]
EN 13726-2:2002	Test methods for primary wound dressings. Part 2: Moisture vapor transmission rate of permeable film dressings	Provides information concerning the evaluation of fluid-handling properties of the wound dressing, which determine the degree of hydration of the wound and surrounding tissues.	[120]
EN 13726-3:2003	Test methods for primary wound dressings. Part 3: Waterproofness	Provides information concerning the evaluation of the ability to prevent strike-through of blood or other fluids.	[121]
EN 13726-4:2003	Test methods for primary wound dressings. Part 4: Conformability	Provides information concerning how comfortable a wound dressing is, measuring resistance to stretching and ability to return to its original shape after stress.	[122]
EN 13726-5:2000 ^1^	Test methods for primary wound dressings. Part 5: Bacterial barrier properties	Provides information concerning the evaluation of the antimicrobial properties of wound dressings.	[123]
EN 13726-6:2003	Test methods for primary wound dressings. Part 6: Odor control	Provides information concerning the evaluation of the efficacy in absorbing odor.	[124]

^1^ Withdrawn in 2012, due to inter-laboratory variability and leakage issues with the test device.

**Table 6:** Summary of the physical characterization of PHMB-releasing membranes (PRMs) developed for antimicrobial dressings (AMDs). Membrane IDs and corresponding bibliographic references are the same as in Table 3. ^1.^

ID	Physical Properties ^2^	Ref.
PRM1	Swelling ratio (PBS): 104 at 25 °C; 6.4 at 50 °C.	[90]
PRM2a	Water uptake capacity (PBS): ca. 900–1200% at 20 °C. MVTR (ASTM E96-90, Procedure D—Water Method): ca. 204 g/m^2^/24 h for all formulations. Air permeability (ASTM D737-04): 2.5–4 cm^3^/cm^2^/s (best: 2:1 blend).	[91]
PRM2b	Water uptake capacity (PBS): ca. 1200–1800% at 20 °C. MVTR (ASTM E96-90, Procedure D—Water Method): ca. 204 g/m^2^/24 h for all formulations. Air permeability (ASTM D737-04): ca. 0.5 cm^3^/cm^2^/s.	
PRM13	Water uptake capacity (SBF): 250% at RT. MVTR (ASTM E96-90, Procedure A—Desiccant Method: ca. 2500 g/m^2^/24 h. Oxygen transmission rate (GB/T 19789-2005): 48 cm^3^/m^2^/12 h.	[103]
PRM15	Water uptake capacity (saline): 233% (temperature not mentioned).	[105]
PRM17	Water uptake capacity (PBS): 2028%; 1379% after sterilization. Equilibrium water content (water): 95% (temperature not mentioned); 93% after sterilization.	[107]
PRM18	Water uptake capacity (PBS): 639% at 37 °C.	[108]
PRM19	Water uptake capacity (PBS): 1051% at 37 °C.	
PRM23a	Water uptake capacity (PBS): 407% at RT.	[112]
PRM23b	Water uptake capacity (PBS): 348% at RT.	
PRM23c	Water uptake capacity (PBS): 353% at RT.	

^1^ PRMs for which their physical characterization was not reported were excluded from this table. ^2^ For consistency purposes and to facilitate comparisons, the terms *water uptake capacity*, *equilibrium water content* and *swelling ratio* were used whenever absorptive capacity was expressed in percent mass increase in relation to dry mass (100% × (swollen sample mass—dry sample mass)/(dry sample mass)), percent mass increase in relation to swollen mass (100% × (swollen sample mass—dry sample mass)/(swollen sample mass)) and swollen to dry mass ratio, respectively. MVTR—Moisture vapor transmission rate; PBS—phosphate-buffered saline; RT—room temperature; SBF—simulated body fluid (composition not provided).

**Table 7:** Summary of the drug-release studies carried out with PHMB-releasing membranes (PRMs) developed for antimicrobial dressings (AMDs). Membrane IDs and corresponding bibliographic references are the same as in Table 3. ^1.^

ID	Drug-Release Assay Conditions (Assay, Release Medium and Sampling Mode)	Type of Release and Duration	[PHMB]_max_ Released
PRM2a	-Batch assay. -Distilled water (40 mL), 37 °C, with shaking. -Sampling not detailed.	-Initial burst release. -ca. 1 h.	np
PRM2b			
PRM3	-Batch assay. -Distilled water (10 mL), 37 °C, with shaking (30 rpm). -2 mL aliquots; replenishment with new medium.	-Initial burst release. -ca. 24 h (for loading with 0.3% PHMB).	np
PRM5	-Batch assay. -50 mL PBS, 37 °C, with shaking (200 rpm). -Aliquots of undisclosed volume; replenishment with new medium.	-Initial burst release. -0.5 h (for loading with 0.25% PHMB).	np
PRM6	-Batch assay. -PBS (3 mL), pH 7.4, at 37 °C; no agitation mentioned. -Sampling not detailed.	-Initial burst release. -ca. 12 h. -Membrane also released SS.	ca. 0.016% *w*/*v*
PRM7	-Batch assay. -Salt solution ^2^ (20 mL), pH 6.6, 32 °C, with shaking (70 rpm). -0.5 mL aliquots; no replenishment.	-Sustained release. -ca. 24 h.	np
PRM8	-Batch assay. -PBS (volume not mentioned), pH 7.4, 37 °C, with stirring. -Sampling: 1.5 mL; replenishment with new medium.	-Bimodal release: 1st burst release for ca. 3 h; 2nd release after 24 h, for 48 h. -Membrane also released SS.	np
PRM10	-Batch assay. -PBS (60 mL), pH 7.4, 37 °C, with shaking (100 rpm). -1 mL aliquots; replenishment with new medium.	-Initial burst release. -4 h (for loading with 1% PHMB) to 11 h (for loading with 10% PHMB).	ca. 0.1–5 µg PHMB/mg of sample
PRM11a	-Single-face release in a cuvette. ^3^ -50 mM TRIS (2 mL), pH 7.4, 37 °C, with unspecified agitation. -Sampling not clear.	-Initial burst release. -ca. 5 h.	ca. 0.002% *w*/*v*
PRM11b		-Bimodal release: 1st burst release for ca. 15 h; 2nd release after ca. 30 h for ca. 20 h.	ca. 0.04% *w*/*v*
PRM13	-Batch assay. -SBF. No further conditions mentioned. -Sampling not detailed.	-Bimodal release: 1st slow release for ca. 15 h; 2nd release for an extra 20 h (for loading with 0.1% PHMB) or 45 h (for loading with 0.2% PHMB) -Highest duration: ca. 60 h (for loading with 0.2% PHMB).	ca. 0.009 (0.1% sample); ca. 0.017% (0.2% sample)
PRM14	-Batch assay. -PBS, RT. No agitation. No other conditions mentioned. -Sampling not detailed.	-Initial burst release followed by sustained release. -ca. 5 days (for loading with 5 and 15 wt% PHMB); 48 h for loading with 25 wt% PHMB); 1 h for loading with 35 wt% PHMB.	From 0.02 to 0.2 mg of PHMB
PRM16	-Batch assay. -PBS (10 mL), pH 7.4, 37 °C, shaking (60 rpm). -2 mL aliquots; replenishment with new medium.	-Initial burst release, followed by sustained release. -ca. 1 day for loading with 1 %wt PHMB; ca. 4 days, for loading with 5 wt% PHMB; ca. 20 days for loading with 10 wt% PHMB.	np
PRM17	-Franz diffusion cell assay. -PBS (volume not mentioned), 34 °C, with stirring. -0.2 mL aliquots; replenishment with new medium.	-Sustained release. -48 h.	4–5 µg PHMB/mg dry sample.
PRM21	-Batch assay. -SBF (1 mL). No further conditions mentioned. -Sampling: 0.3 mL sample; no further details.	-Burst release for loading with 0.3% PHMB; duration: ca. 24 h. -Bimodal release for loading with 0.1 and 0.5 wt% PHMB: 1st release for ca. 24 h; 2nd release after 24 h, for another 24 h.	ca. 0.02%–0.05% *w*/*v*
PRM22a	-Batch assay. -PBS. No further details. -Sampling: not mentioned.	-Initial burst release. -Duration: ca. 48 h (for loading with 5% PHMB).	np
PRM22b		-Small initial burst release, followed by sustained release. -Duration: ca. 5 days (for loading with 5% PHMB in cationic nanoliposomes).	
PRM23a	-Batch assay. -PBS (50 mL), pH 7.4, 37 °C, with stirring (50 rpm). -5 mL aliquots; replenishment with new medium.	-Initial burst release. -Duration: 4 h.	np

^1^ PRMs not studied or for which a plot of the released drug versus time was not in the published report were excluded from this table. ^2^ NaCl 8.6 g/L, KCl 0.3 g/L and CaCl_2_·2H_2_O 0.33 g/L. ^3^ Sample prepared at the bottom of a cuvette. np—Not provided. Max—Maximum; PBS—phosphate-buffered saline; RT—room temperature; SBF—simulated body fluid (composition not provided); SS—silk sericin; TRIS—tris(hydroxymethyl)aminomethane.

**Table 8:** In vitro antimicrobial activity evaluation of PHMB-releasing membranes (PRMs) developed for antimicrobial dressings (AMDs). Membrane IDs and corresponding bibliographic references are the same as in Table 3. ^1.^

ID	PHMB Loading	Antimicrobial Activity Evaluation (Test Organism, Assay and Results)
PRM1	Soaking. [PHMB]: 0.1 or 1% *w*/*v*	-*E. coli* -Test bacteria inoculated into aliquots collected from a PBS solution 1, 2, 3, 4, 5 and 6 h after the immersion of the membrane, followed by overnight incubation at 37 °C. ■ Very significant reduction in bacterial numbers at all times tested, peaking at 2 h. **Note:** This PRM was also assayed for infection control, employing a rat full-thickness excisional wound model infected with *P. aeruginosa*, at days 4, 8 and 12 post-surgery. Decreases in surface bacterial population were significantly more pronounced for the treated groups (unloaded and loaded membranes) than for the untreated control for samples collected at days 8 and 12. No differences in deep tissue bacterial population between treated and untreated groups for all time points.
PRM2a	Addition. [PHMB]: 1 wt%	-*E. coli* -Test bacteria inoculated into PRM, followed by 5 h incubation at 37 °C. ■ High antibacterial activity; almost complete bacterial elimination.
PRM2b		-*E. coli* -Test bacteria inoculated into PRM, followed by 5 h incubation at 37 °C. ■ High antibacterial activity; almost complete bacterial elimination.
PRM3	Addition. [PHMB]: 0.15 or 0.3% *w*/*v*	*-S. aureus* and *E. coli* -PRM applied to an agar plate with test bacteria (agar disc diffusion assay), followed by 24 h incubation at 36 °C. ■ High, [PHMB]-dependent antibacterial activity for both test bacteria.
PRM5	Addition. [PHMB]: 0.02, 0.075, 0.15 or 0.25% *w*/*v*	-*E. coli* and *M. luteus* -Test bacteria inoculated into PRM, followed by 48 h incubation at 37 °C. ■ Total growth inhibition of both bacteria for PRMs loaded with [PHMB] ≥ 0.15%. PRMs loaded with 0.02 and 0.075% *w*/*v* PHMB did not kill bacteria, even after 45 h.
PRM6	Impregnation. [PHMB]: 0.3% *w*/*v*	-*B. subtilis*, *S. aureus*, MRSA, *E. coli*, *A. baumannii*, and *P. aeruginosa* -PRM applied to an agar plate with test bacteria (agar disc diffusion assay), followed by 24 h incubation at 37 °C. -Test bacteria inoculated into PRM, followed by 24 h incubation at 37 °C. ■ High antibacterial activity against all bacteria in both assays. ■ Agar disc diffusion assay: antibacterial activity was superior to a commercial CHX-based AMD (Bactigras^®^); comparable to a commercial silver-based AMD (Acticoat^®^) for Gram-negative bacteria, but inferior against *P. aeruginosa*; comparable to a commercial PRWD (Suprasorb^®^ X + PHMB), but inferior against *B. subtilis*.
PRM7	Soaking. [PHMB]: 1% *w*/*v*	-*S. aureus* -Test bacteria inoculated into PRM and into PRM extracts prepared in CB medium (0.02–20 mg membrane/mL DMEM; 24 h at 37 °C), followed by 24 h incubation at 37 °C. ■ High antibacterial activity observed for PRM and for extraction ratios > 0.02 mg membrane/mL medium.
PRM8	Soaking. [PHMB]: 0.2% *w*/*v*	-*E. coli, A. baumannii, P. aeruginosa, B. subtilis, S. aureus* and MRSA -PRM applied to an agar plate with test bacteria (agar disc diffusion assay), followed by 24 h incubation at 37 °C. ■ Active against all bacteria, except *P. aeruginosa*. Activity comparable to that of an equivalent membrane loaded with PVP-I. ■ Activity against *E. coli, A. baumannii, B. subtilis* and *S. aureus*, but not against MRSA, lost upon storage of PRM at 30 °C for 1–6 months. A commercial WD (Allevyn^®^) maintained its antibacterial activity for 6 months of storage.
PRM9	Addition. [PHMB]: np	-MSSA, *P. aeruginosa* and *K. pneumoniae* -PRM applied to an agar plate with test bacteria (agar disc diffusion assay), followed by repeated 24 h incubations for 7 days (temperature not mentioned). ■ Antibacterial activity lasted for 24 h only.
PRM10	Addition. [PHMB]: 1 or 10 wt%	-*S. aureus* and *P. aeruginosa* -PRM applied to an agar plate with test bacteria (agar disc diffusion assay), followed by 48 h incubation at 35 °C. -Test bacteria inoculated into PRM on an agar plate, followed by 48 h incubation at 35 °C. ■ Antibacterial activity only for [PHMB] = 10 wt% PHMB. No activity against *P. aeruginosa*. Bacterial permeation through the PRM occurred. No bacterial growth on the PRM surface.
PRM11a	Addition. [PHMB]: 0.4% *w*/*v*	-*P. aeruginosa* and *S. aureus* as colonies on agar plates and as single species biofilms -PRM applied to an agar plate with test bacteria (agar disc diffusion assay), followed by 48 h incubation at 37 °C. -PRM applied to single-species biofilms on glass coverslips, followed by 24 h incubation at 37 °C. ■ Agar disc diffusion assay: high antibacterial activity observed; not due to PHMB but to another PRM component (EDTA). ■ Biofilm assay: high reduction in the number of viable bacteria after 24 h. Lower activity against *S. aureus*.
PRM11b	Addition. [PHMB]: 0.4% *w*/*v*	-*P. aeruginosa* e *S. aureus* as colonies on agar plates and as single- and as multi-species biofilms -PRM applied to an agar plate with test bacteria (agar disc diffusion assay), followed by 48 h incubation at 37 °C. -PRM applied to biofilms on glass coverslips, followed by 24 h incubation at 37 °C. ■ Agar disc diffusion assay: high antibacterial activity observed; not due to PHMB but to another PRM component (EDTA). ■ Biofilm assay: reduction in the number of viable bacteria in single- and multi-species biofilms, after 24 h; less active against single-species biofilms of *S. aureus*. Total elimination of all single- and mixed-species biofilms after 48 h.
PRM12	Impregnation. [PHMB]: 0.04% or 0.1% (+0.1% UDAPB)	-*S. aureus* -PRM applied to an agar plate with test bacteria (agar disc diffusion assay), followed by overnight incubation at 37 °C. ■ Antibacterial activity comparable to that of a commercial PRWD (Suprasorb^®^ X + PHMB) for PRMs loaded with both [PHMB], although the PRWD was loaded with a [PHMB] that was 7.5- or 3-times higher, respectively.
PRM13	Soaking. [PHMB]: 0.1 or 0.2% *w*/*v*	-*S. aureus* and *E. coli* -PRM applied to an agar plate with test bacteria (agar disc diffusion assay), followed by 24 h incubation at 37 °C. -Test bacteria inoculated into PRM, followed by 24 h incubation at 37 °C. -Fungal growth assay: sample storage in a humid environment. ■ Agar disc diffusion assay: high activity against both bacteria; higher activity for loading with 0.2% *w*/*v* PHMB. ■ Inoculation assay: almost no bacteria survived on the PRM’s surface. ■ Fungal growth assay: no fungal growth for at least 6 weeks.
PRM14	Addition. [PHMB]: 5, 15, 25 or 35% wt% relative to polymer mass	-*S. aureus* -PRM applied to an agar plate with test bacteria (agar disc diffusion assay), followed by overnight incubation at 37 °C. -Test bacteria inoculated into PRM, followed by overnight incubation at 37 °C. ■ Agar disc diffusion assay: antibacterial activity increased with [PHMB], with highest activities for [PHMB] ≥ 15%. Superior to a commercial silver-based AMD (Actisorb^®^ Silver 220), that did not show antibacterial activity. ■ Inoculation assay: no surviving bacteria after 24 h for [PHMB] ≥ 15%. Superior to Actisorb^®^ Silver 220 that showed minimal antibacterial activity.
PRM15	Soaking. [PHMB]: 0.1, 0.5, 1 or 2% *w*/*v*	-*K. pneumoniae*, *A. baumannii*, and *S. aureus* -PRM applied to an agar plate with test bacteria (agar disc diffusion assay), followed by repeated 24 h incubations for 9 days at 35 °C. ■ Antibacterial activity up to 3–5 days for PRMs loaded with 0.1 and 0.5% PHMB and up to 7–8 days for PRMs loaded with 1 and 2% PHMB. **Note**: This PRM ([PHMB]: 1% *w*/*v*) was also assayed for infection control in a rat full-thickness excisional wound model infected with *A. baumannii*., at days 3, 7, 14 and 21 post-surgery. A decrease in wound infection was observed during the earlier treatment stage. After 21 days, bacteria were still present, but in significantly lower numbers than in the control group (unloaded membrane); however, 3 out of 10 animals still showed infection symptoms.
PRM16	Addition. [PHMB]: 0.5, 1, 2, 5, 10 wt% relative to polymer mass	-*S. aureus* and *E. coli* -PRM applied to an agar plate with test bacteria (agar disc diffusion assay), followed by 24 h incubation at 37 °C. ■ Antibacterial activity for PRMs loaded with [PHMB] ≥ 2 wt%, with high activity for [PHMB] of 5 and 10 wt%.
PRM17	Soaking. [PHMB]: 0.5% *w*/*v*	-*S. aureus* and *S. epidermidis* -PRM applied to an agar plate with test bacteria (agar disc diffusion assay), followed by 24 h incubation at 37 °C. ■ Antibacterial activity against both bacteria.
PRM18	Addition. [PHMB]: 0.04, 0.08, 0.12, 0.16 or 0.2% *w*/*v*	-*S. aureus* -PRM applied to an agar plate with test bacteria (agar disc diffusion assay), followed by 24 h incubation at 36 °C. ■ Antibacterial activity for all [PHMB]. Antibacterial activity also due to AgNPs. Optimal [PHMB]: 0.12% *w*/*v*. ■ PRM19 with higher antibacterial activity than PRM18.
PRM19		
PRM20	Impregnation. [PHMB]: 0.1% *w*/*v* (+0.1% *w*/*v* UDAPB)	-*S. aureus*, *S. epidermidis*, *E. faecium*, *E. coli*, *K. pneumoniae*, *E. cloacae*, *P. aeruginosa*, *A. baumannii*, and the fungal species *C. albicans* as biofilm cultures -PRM applied to an agar plate with test bacteria (agar disc diffusion assay), followed by overnight incubation at 37 °C. -PRM contacted with a biofilm on agar discs, followed by 24 h incubation at 37 °C. ■ Agar disc diffusion assay: inhibition of growth of all bacterial strains, in contrast to a commercial silver-based AMD (Aquacel^®^ Ag). Less effective for most bacterial strains than equivalent membranes loaded with PVP-I (7.5%) and CHX (0.5%). ■ Contact with biofilms assay: reduced bacterial growth of all tested bacterial strains in AE and TSB media, with the exception of a single strain of *E. epidermidis* in TSB. Better performance than Aquacel^®^ Ag, but slightly inferior performance than equivalent membranes loaded with OCT and PVP-I. Activity depended on the growth medium employed, being higher in the AE medium.
PRM21	Addition. [PHMB]: 0.1, 0.3 or 0.5 wt%	-*P. aeruginosa*, *A. baumannii*, *S. aureus*, *S. epidermidis*, *S. pyogenes*, *B. subtilis* and *C. albicans* -PRM applied to an agar plate with test bacteria (agar disc diffusion assay), followed by 18 h incubation at 37 °C. -Test bacteria inoculated into PRM, followed by 24 h incubation at 36 °C. ■ Agar disc diffusion assay: antibacterial activity with almost all [PHMB] concentrations against almost all species. The exceptions were the absence of activity against *P. aeruginosa* and *A. baumannii* for all [PHMB] and against *B. subtilis* for 0.1 and 0.5 wt% PHMB. ■ Inoculation assay: inhibition of biofilm formation of all bacterial species increased with [PHMB], being higher for PRMs loaded with 0.5 wt% PHMB, except *B. subtilis*, which was not inhibited.
PRM22a	Soaking. [PHMB]: 0.2, 0.5, 1, 2, 3 or 5% *w*/*v*	-*E. coli* and *S. aureus* -Test bacteria inoculated into PRM, followed by 1 or 3 h incubation (temperature not mentioned). ■ High antibacterial activity for PRMs loaded with [PHMB] ≥ 0.5%.
PRM22b	Soaking. [PHMB]: 0.2, 0.5, 1, 2, 3 or 5% *w*/*v* (in NLs)	-*E. coli* and *S. aureus* -Test bacteria inoculated into PRM, followed by 1 or 3 h incubation (temperature not mentioned). ■ High antibacterial activity for PRMs loaded with [PHMB] ≥ 0.5%.
PRM23a	Soaking. [PHMB]: nc	-*S. aureus* and *P. aeruginosa* -PRM applied to an agar plate with test bacteria (agar disc diffusion assay), followed by overnight incubation at 37 °C. ■ High antibacterial activity against both bacteria.

^1^ PRMs not evaluated were excluded from this table. AE—Artificial exudate (1% of mucin, 1% of bovine serum albumin, 10% of fetal bovine serum and 88% of RPMI); *A. baumannii*—*Acinetobacter baumannii*; AgNP—silver nanoparticles; AMD—antimicrobial dressing; *B. subtilis*—*Bacillus subtilis*; *C. albicans*—*Candida albicans*; CB—Caso-Bouillon; CHX—chlorhexidine; DMEM—Dulbecco’s Modified Eagle Medium; *E. cloacae*—*Enterobacter cloacae*; *E. coli*—*Escherichia coli*; EDTA—ethylenediaminetetraacetic acid; *E. faecium*—*Enterococcus faecium*; *K. pneumoniae*—*Klebsiella pneumoniae*; *M. luteus*—*Micrococcus luteus*; MRSA—methicillin-resistant *S. aureus*; MSSA—methicillin-sensitive *S. aureus*; NL—nanoliposome; OCT—octenidine; *P. aeruginosa*—*Pseudomonas aeruginosa*; PBS—phosphate-buffered saline; PRWD—PHMB-releasing wound dressing; PVP-I—povidone-iodine; *S. aureus*—*Staphylococcus aureus*; *S. epidermidis*—*Staphylococcus epidermidis*, *S. pyogenes*—*Streptococcus pyogenes*; TSB—tryptic soy broth; UDAPB—undecylenamidopropyl betaine.

**Table 9:** Cytotoxicity evaluation of PHMB-releasing membranes (PRMs) developed for antimicrobial dressings (AMDs). Membrane IDs and corresponding bibliographic references are the same as in Table 3. ^1.^

ID	PHMB Loading	Cytotoxicity Evaluation (Sample Tested, Cell Model, Contact Type, Contact Duration, Endpoint, Assay and Results)
PRM2b	Addition. [PHMB]: 1 wt% relative to polymer mass	-Membrane. -Rat skin fibroblasts. -Cells seeded on membrane, followed by 3-day incubation. -Percentage of live cells (to total number of cells) and cell morphology (microscopy). ■ No cytotoxicity. ■ No cytotoxicity for unloaded membrane.
PRM5	Addition. [PHMB]: 0.02, 0.075, 0.15 and 0.25% (PHMB weight to formulation volume)	-Membrane. -MDCK epithelial canine kidney cells and MRC-5 human embryo lung fibroblasts. -Cells seeded on membrane, followed by 4-day incubation (cell viability). -Cell viability, as assessed by oxidoreductase activity (MTT assay). ■ Cytotoxicity for 0.25% *w*/*v* PHMB against both cell lines. ■ No cytotoxicity for unloaded membrane.
PRM6	Impregnation. [PHMB]: 0.3% *w*/*v*	-Membrane extracts prepared in cell culture medium (1 cm^2^ membrane/3 mL DMEM; 24 h). -L929 mouse subcutaneous fibroblasts. -Extracts added to cells, followed by 24 h incubation. -Cell viability, as assessed by oxidoreductase activity (MTT assay). ■ No cytotoxicity. ■ No cytotoxicity for Bactigras^®^, a commercial CHX-based AMD, tested under the same conditions.
PRM7	Soaking. [PHMB]: 1% *w*/*v*	-Membrane extracts prepared in cell culture medium (0.02–20 mg membrane/mL DMEM; 24 h at 37 °C). -HaCaT human epidermal keratinocytes. -Extracts added to cells, followed by 1, 24 and 48 h incubations. -Cell proliferation, as assessed by ATP levels (ATPLite^TM^-M assay). ■ Incubation time- and extraction ratio-dependent effects. Cytotoxicity at 24 h for extraction ratios ≥ 2 mg membrane/mL DMEM. ■ No cytotoxicity for unloaded membrane.
PRM14	Addition. [PHMB]: 5, 15, 25 and 35 wt% relative to polymer mass	-Membrane. -HaCaT human epidermal keratinocytes. -Membrane placed in direct contact with the cell monolayer, followed by 24 and 48 h incubations. -Cell viability, as assessed by oxidoreductase activity (alamarBlue^TM^ assay). ■ Cytotoxicity for 15, 25 and 35 wt% PHMB for both time points. ■ No cytotoxicity for unloaded membrane. ■ Cytotoxicity for Actisorb^®^, a commercial silver-based AMD, tested under the same conditions.
PRM15	Soaking. [PHMB]: 1% *w*/*v*	-Membrane. -Human skin fibroblasts (CI 0159) and human vascular endothelial cells (CI 0478). -Cells seeded on membrane, followed by 3-, 7-, 14- and 21-day incubations. -Cell viability, as assessed by oxidoreductase activity (MTT assay). ■ No cytotoxicity. ■ No cytotoxicity for unloaded membrane.
PRM17	Soaking. [PHMB]: 0.5% *w*/*v*	-Membrane. -NIH 3T3 mouse embryo fibroblasts. -Membrane placed on hanging inserts above cells (indirect contact), followed by 24 h incubation. -Cell viability, as assessed by oxidoreductase activity (MTT assay). ■ No cytotoxicity.
PRM21	Addition. [PHMB]: 0.1, 0.3 or 0.5 wt% relative to solution mass	-Membrane extracts prepared in cell culture medium (4 cm^2^ membrane/2 mL DMEM; 24 h at 37 °C). -L929 mouse subcutaneous fibroblasts and HaCaT human epidermal keratinocytes. -Extracts added to cells, followed by 3-day incubation. -Cell viability, as assessed by oxidoreductase activity (MTT assay). ■ [PHMB]-dependent decrease in cell viability for both cell lines. Cytotoxicity for 0.5 wt% PHMB, but only against HaCaT cells. ■ No cytotoxicity for unloaded membrane extract.
PRM22a	Soaking. [PHMB]: 1 or 5% *w*/*v*	-Membrane extracts prepared in cell culture medium (6.5 cm^2^ membrane/2 mL DMEM; 24 h). -Primary human skin fibroblasts. -Extracts added to cells, followed by 24 h incubation. -Cell viability, as assessed by oxidoreductase activity (MTT assay). ■ Mild cytotoxicity for both PHMB concentrations. ■ No cytotoxicity for unloaded membrane extract.
PRM22b	Soaking. [PHMB]: 1 or 5% *w*/*v*, encapsulated in nanoliposomes	-Membrane extracts prepared in cell culture medium (6.5 cm^2^ membrane/2 mL DMEM; 24 h). -Primary human skin fibroblasts. -Extracts added to cells, followed by 24 h incubation. -Cell viability, as assessed by oxidoreductase activity (MTT assay). ■ Subcytotoxicity (cell viabilities of ca. 70% for both PHMB concentrations). ■ No cytotoxicity for unloaded membrane extract.
PRM23a	Soaking. [PHMB]: nc	-Membrane extracts prepared in cell culture medium (2 × 2 × 2 mm^3^ in DMEM; 48 h). -HaCaT human epidermal keratinocytes. -Extracts added to cells, followed by 24 h incubation. -Cell viability, as assessed by oxidoreductase activity (MTT assay). ■ No cytotoxicity. ■ No cytotoxicity for unloaded membrane extract.

^1^ PRMs not evaluated were excluded from this table. nc—Not clear. ATP—Adenosine triphosphate; CHX—chlorhexidine; DMEM—Dulbecco’s Modified Eagle Medium; MTT—3-(4,5-dimethylthiazol-2-yl)-2,5-diphenyltetrazolium bromide.

**Table 10:** Additional biological effects evaluated with PHMB-releasing membranes (PRMs) developed for antimicrobial dressings (AMDs). Membrane IDs and corresponding bibliographic references are the same as in Table 3.

ID	Other Biological Studies
PRM6	-Subcutaneous implantation in rats, followed for 28 days. ■ Lower inflammatory response than with a CHX-based commercial AMD. -Sensitization evaluation in healthy volunteers, by a patch test, with 3 patches applied consecutively for 9 days. ■ Non-sensitizing.
PRM15	-Intradermal stimulation in rabbits, by injection of aliquots from the drug-release study. Observed for 72 h. ■ Minimal intradermal stimulation. -Sensitization in guinea pigs, by a patch test, for 21 days. ■ No sensitization. -Subcutaneous implantation in rats, for 21 days. ■ Good healing. No infection. No necrosis.
PRM17	-HET-CAM irritation test with 5 min of contact. ■ No irritation.
PRM18	-Hemostatic capacity qualitatively evaluated through visual inspection of blood added to the WDs. ■ Alginate increased blood-clotting rate.

AMD—Antimicrobial dressing; CHX—chlorhexidine; HET-CAM: hen’s egg test—chorioallantoic membrane; WD—wound dressing.

**Table 11:** In vitro and in vivo evaluation of wound-healing capacity of PHMB-releasing membranes (PRMs) developed for antimicrobial dressings (AMDs). Membrane IDs and corresponding bibliographic references are the same as in Table 3. ^1.^

ID	Wound-Healing Evaluation (Model, Controls, Duration, Parameters Evaluated and Results)
PRM1	-Model: rat full-thickness excisional wound model. -Controls: untreated wound and wound treated with unloaded membrane. -Assay duration: 12 days. -Parameter evaluated: wound size. -Results: only the sample loaded with 0.1% PHMB was evaluated. Decreased wound size for treatment and unloaded membrane groups relative to the untreated control for all time points studied. After 12 days, most wounds were closed.
PRM2b	-Model: rat burn wound model. -Controls: untreated wound and wound treated with unloaded membrane. -Assay duration: 28 days. -Parameter evaluated: observation of the wound and histological evaluation. -Results: the best formulation showed an almost regenerated epidermal layer, while control groups showed an ongoing inflammatory response.
PRM4	-Case study: two human diabetic toe amputation wounds. -Results: complete wound healing in 4 and in 60 days.
PRM6	-Model: rat full-thickness excisional wound model. -Controls: wound treated with a CHX-based AMD. -Assay duration: 21 days. -Parameters evaluated: wound size and area covered by collagen, after staining. -Results: after 14 days, higher reduction in wound size and higher collagen formation than with a commercial CHX-based AMD. After 21 days, wound almost fully closed, in contrast to wound treated with the CXH-based AMD treatment. -In vitro evaluation: wound-scratch assay. ■ Assay: wound-scratch. ■ Model: L929 mouse fibroblasts. ■ Sample: extracts in cell culture medium (DMEM). ■ Controls: extract of commercial CHX-based AMD and DMEM. ■ Assay duration: 72 h. ■ Result: cell migration comparable to the DMEM control and to the commercial CHX-based AMD. -In vitro evaluation: Boyden’s chamber assay. ■ Assay: Boyden’s chamber. ■ Model: L929 mouse fibroblasts. ■ Sample: extracts in DMEM. ■ Controls: extract of commercial CHX-based AMD in DMEM and DMEM. ■ Assay duration: 24 h. ■ Results: cell migration comparable to the DMEM control and to the commercial CHX-based AMD.
PRM13	-Model: rat incisional wound model -Controls: untreated wound and wound treated with 2 commercial WDs (not AMDs). -Assay duration: 4 weeks, with weekly changes. -Parameter evaluated: wound area. -Results: only the sample loaded with 0.2% PHMB was evaluated. Faster decrease in wound size for treatment with the PRM compared to the untreated group and to the 2 groups treated with the 2 commercial WDs, being the only one that showed a fully closed wound after 4 weeks.
PRM15	-Model: rat full-thickness excisional wound model, with sample sutured to the wound. -Controls: treatment with unloaded membrane. -Assay duration: over a 21-day period. -Parameters evaluated: angiogenesis and inflammatory indicators. -Results: only the 1% PHMB sample was evaluated. No effect on angiogenesis or on inflammatory indicators. No effect on neovascularization or wound-repair capacity.
PRM17	-Case study: dog with contact ulcers on rear limbs. -Results: complete healing after 3 weeks of treatment, in contrast to wound treated with a non-medicated commercial WD.

^1^ PRMs for which a wound-healing evaluation was not reported were excluded from this table. AMD—Antimicrobial wound dressing; CHX—chlorhexidine; DMEM—Dulbecco’s Modified Eagle Medium; WD—wound dressing.

## Data Availability

Not applicable.
